# The Challenges and Achievements of Experimental Implementation of an Active Transfemoral Prosthesis Based on Biological Quasi-Stiffness: The CYBERLEGs Beta-Prosthesis

**DOI:** 10.3389/fnbot.2018.00080

**Published:** 2018-12-04

**Authors:** Louis Flynn, Joost Geeroms, Rene Jimenez-Fabian, Sophie Heins, Bram Vanderborght, Marko Munih, Raffaele Molino Lova, Nicola Vitiello, Dirk Lefeber

**Affiliations:** ^1^Department of Robotics and Multibody Mechanics, Vrije Universiteit Brussel, and Flanders Make, Brussels, Belgium; ^2^Center for Research in Mechatronics, Institute of Mechanics, Materials, and Civil Engineering, Institute of Neuroscience, and Louvain Bionics, Université Catholique de Louvain, Louvain-la-Neuve, Belgium; ^3^Robolab, Faculty of Electrical Engineering, University of Ljubljana, Ljubljana, Slovenia; ^4^Fondazione Don Carlo Gnocchi, Milan, Italy; ^5^The BioRobotics Institute, Scuola Superiore Sant'Anna, Pisa, Italy

**Keywords:** prosthesis, transfemoral, active, knee, ankle, powered, quasi-stiffness, compliant

## Abstract

The CYBERLEGs Beta-Prosthesis is an active transfemoral prosthesis that can provide the full torque required for reproducing average level ground walking at both the knee and ankle in the sagittal plane. The prosthesis attempts to produce a natural level ground walking gait that approximates the joint torques and kinematics of a non-amputee while maintaining passively compliant joints, the stiffnesses of which were derived from biological quasi-stiffness measurements. The ankle of the prosthesis consists of a series elastic actuator with a parallel spring and the knee is composed of three different systems that must compliment each other to generate the correct joint behavior: a series elastic actuator, a lockable parallel spring and an energy transfer mechanism. Bench testing of this new prosthesis was completed and demonstrated that the device was able to create the expected torque-angle characteristics for a normal walker under ideal conditions. The experimental trials with four amputees walking on a treadmill to validate the behavior of the prosthesis proved that although the prosthesis could be controlled in a way that allowed all subjects to walk, the accurate timing and kinematic requirements of the output of the device limited the efficacy of using springs with quasi-static stiffnesses. Modification of the control and stiffness of the series springs could provide better performance in future work.

## 1. Introduction

Current transfemoral prostheses are most often passive, modular systems that cannot generate joint work. From fully passive ankles such as the SACH foot (Staros, 1957), Energy-Storage-Return (ESR) (Hafner et al., 2002) carbon types, and fully passive knees, such as the Mauch knee (Mauch, 1968), to current top of the line microcontroller knee prostheses (Otto Bock Genium and C-Leg, Ossur Rheo Knee, Blatchford/Endolite Pleo, Freedom Innovations Plié, among others), there are no systems that provide additional output energy; the only energy used for prosthesis propulsion is energy that has been captured from the gait cycle.

Providing positive work is an important aspect of the biological joint and there are new robotic designs that are capable of delivering it. Devices such as the OttoBock emPOWER (previously iWalk, BionX, BiOM Au and Herr, 2008), or the Össur/Springactive Odyssey (Hitt et al., 2008) ankles or the Össur Power Knee are available, or will soon be available, as commercial devices. While not widely prescribed at the moment, they are beginning to find use in the market. There are many reasons for believing that active ankle and knee propulsion provides benefit such as tests which have shown a reduction in loading of the unaffected leg using a powered ankle (Grabowski and D'Andrea, 2013), reduction of the metabolic energy consumption of a transtibial amputee to the level of a non-amputee while using robotic ankles (Herr and Grabowski, 2012; Caputo and Collins, 2014), and simulations showing reductions in metabolic cost below normal human walking (Handford and Srinivasan, 2016).

A major drawback of all of these active propulsion systems is the high electrical demand and increased actuator complexity of providing such work. This has led to a large variety in the designs of robotic prostheses, mainly differing from how passive compliance elements are used within the system in order to reduce total energy consumption and motor requirements. Representing the fully active design principle, the Vanderbilt prosthesis only contains one spring, a 6 Nm/deg parallel spring (Lawson et al., 2014) in the ankle that works during the plantarflexion stage of the gait cycle. The operation of this device is fully driven through the use of motors and harmonic drives, requiring the use of impedance controllers to create compliant prosthesis behaviors. The CSEA (Rouse et al., 2014) knee uses a simple friction clutch to lock a series-elastic spring element which passively replicates the torque-angle characteristics during the portion of the gait cycle directly after heel strike. Outside of locking during this short period, the knee is driven with a stiff SEA. This is similar to the clutch and SEA arrangement in the Össur Power knee, which utilizes a dog clutch behind the harmonic drive which is capable of performing the same type of function (Gilbert and Lambry, 2013), although it is unclear if the clutch is used in this manner. Both of these devices have spring stiffnesses chosen to approximate the biological knee quasi-stiffness during the early stance phase of the gait cycle. The ETH/Delft knee (ANGELAA) spent fine attention to the arrangement of parallel and series elastic elements to passively match the actuator stiffness to the desired actual joint stiffness based on simulation and gait studies (Pfeifer et al., 2014). This allows a passive minimization of actuator work necessary to provide desired output impedance.

These devices all have added mechanical complexity and require additional control techniques to accurately detect gait and activate control when compared to their passive equivalents. The RIC Hybrid Knee Prostheses avoid some of this actuator complexity by removing the influence of the actuator on the normal gait cycle. The devices consists of a passive mechanical knee that is used for most normal walking conditions, but also have a motor that can be engaged and disengaged using a clutch (Lenzi et al., 2015) or a transmission (Lenzi et al., 2018) when the actuator is required, such as for sit-to-stand and stair climbing operations.

**Figure 1:**
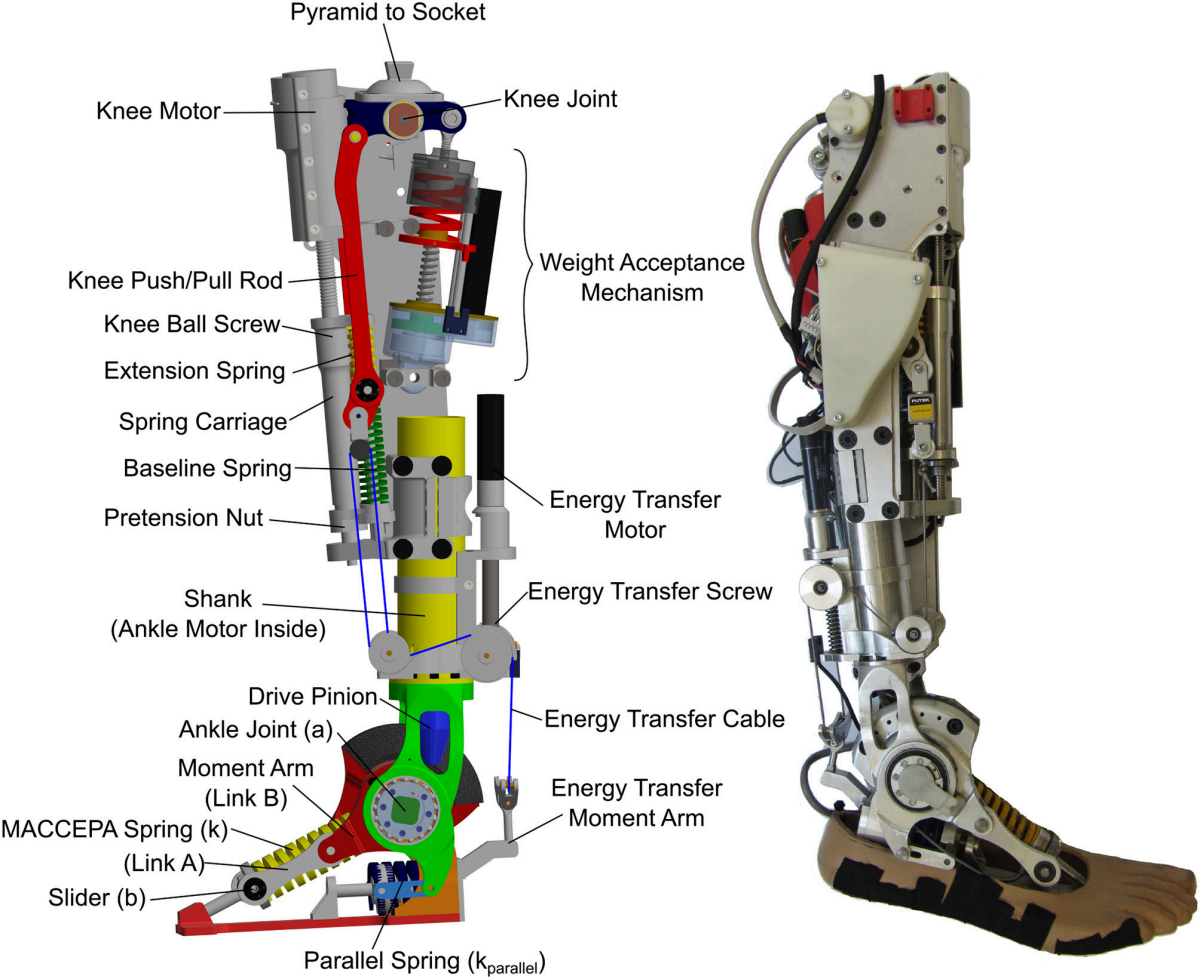
The CYBERLEGs Beta-Prosthesis. Left is a CAD model with all of the relevant components labeled. The front of the knee shows the components of the knee drive including the carriage and series elastic springs. The WA and ET mechanisms can be found at the back of the knee. Right is the realized prosthesis with the electronics and Energy Transfer module connected in the locked position.

Here we have designed an active prosthesis, the CYBERLEGs Beta-Prosthesis (Figure 1), that uses compliant springs whose stiffnesses correspond to the quasi-stiffness of the knee and ankle, which results in relatively soft series spring values. These soft springs theoretically reduce the energy consumption for the motors during normal walking by minimizing motor output work (Geeroms et al., 2017, 2018), but can cause issues for motor controller techniques due to the reduction of actuator bandwidth associated with low output stiffness. The intention is to determine if passively following these quasi-stiffness behaviors with a position based state machine controller provides normal level ground walking capability with low actuator effort while remaining controllable enough for individuals to ambulate and perform other tasks.

The knee torque angle characteristics were divided into three gait regions, and using separate systems that were optimized for particular portions of the gait cycle. The main knee actuator is a highly compliant series elastic actuator, which has the ability to change knee energy during all periods of the gait cycle. There is a Weight Acceptance (WA) mechanism to efficiently handle stance flex in the knee directly after heel strike. Connecting the knee and ankle is a special Energy Transfer (ET) mechanism, a system built with the intention of utilizing captured work from the knee to assist the ankle motor in driving the ankle. This has been explored before in passive devices (Matthys et al., 2012; Unal et al., 2013), but never in an active prosthesis. The combination of systems of the CYBERLEGs Beta-Prosthesis creates a highly passive system for normal level ground walking while remaining capable of providing the high torques and power output for high energy output tasks. The result is a device that has a unique mix of passive and active capabilities, allowing efficient locomotion through passive behaviors, but is capable of actively driving the joints when necessary. For a summary of other tasks such as sit-to-stand, obstacle avoidance, and stair climbing capabilities that were attempted with this device, please refer to Flynn et al. (2018).

### 1.1. Article Contribution

Here we discuss the development of the CYBERLEGs Beta-Prosthesis, the design of which requires four major systems to work together to produce the desired joint torque/angle output. This device was first tested on the bench and found to reduce motor energy consumption while generating expected torque-angle characteristics for a normal walker under ideal conditions.

In amputee trials, four individuals were able to walk over level ground with the prosthesis using a simple state machine based controller. Because of the requirements of the complex relationships of the four major systems, the output kinematics, and the behavior of the person using the device, the position based control technique was not capable of producing the desired output kinematics rendering the ET system ineffective. In general, the use of the quasi-stiffness to determine series actuator spring stiffness with a controller using position setpoints can reduce the motor electrical energy consumption as long as the output kinematics, and therefore the joint torques, are near normal. In actual use, people do not find a way to use the device in a way that provides these natural kinematics, and therefore the position targets must be changed allowing people to walk, but reducing the efficacy of the series elastic actuators due to the compensation for deviations in normal torque/angle characteristics.

This paper defines each of the different systems that are contained within the CYBERLEGs prosthesis, first describing the design rationale, desired behavior, and solutions (section 2). The experiments run on the bench and in subject trials are described in section 3. Results of the behavior of the prosthesis during bench (section 4.2) and amputee validation testing (section 4.3) are then presented. We then discuss the results (section 5) and conclude with future work planned for the prosthesis system (section 6).

## 2. Materials and Methods

The Beta-Prosthesis is a transfemoral prosthesis that contains an active drive in both the knee and the ankle that are both capable of net power output on each of the joints in the sagittal plane. The design began as a passive knee/active ankle system in the Alpha-Prosthesis (Flynn et al., 2015) and had many new concepts added, particularly an entirely new knee system that allowed for net positive work actuation at torques higher than normal walking as well as keeping the passive elements that were demonstrated to work in the Alpha-Prosthesis.

### 2.1. Development of the Beta-Prosthesis

The CYBERLEGs Prosthesis was created as a part of the CYBERLEGs FP7-ICT Project, which combines a prosthesis system to replace a lost limb in parallel with an exoskeleton to assist the sound leg (Giovacchini et al., 2015), and sensory array to control both systems (Goršic et al., 2014). The end goal of the CYBERLEGs system was to assist those who have both a loss of a limb and weakness in the remaining limb to regain walking function and improve walking behavior. Integration within this complete system had influence on the design of the device, particularly in control and electronics architecture.

The CYBERLEGs Beta-Prosthesis consists of four major systems (Figure 1) that when combined can reproduce the knee and ankle torque and kinematics for the knee and ankle for normal walking as determined by biological data. The first system is a powered ankle based on a MACCEPA architecture which gives the ankle a very low stiffness around the neutral position and quickly stiffens as the ankle is displaced from the neutral position. The ankle is driven by a 200 W motor capable of high net power output. The ankle has an added parallel spring to change the passive stiffness of the ankle, to assist the drive and reduce the peak torque required of it. The second system is the Weight Acceptance (WA) system. This is a simple spring that is inserted at the knee during early stance phase to provide the natural characteristics of stance flex without powered actuation. The WA system is capable of producing large reaction torques as external torques are applied, removing the need for the main actuator to operate during early stance and greatly reducing energy consumption. The third system is the Knee Drive Baseline Actuator (KD). This actuator is the main positive energy source of the knee joint, but under nominal use is primarily used to hold the Baseline Spring (BL) in place. During the gait cycle it is possible to fully drive the knee using this system, and it provides all of the power for sit-to-stand and stair climbing operations. The fourth system is the ET system. This system provides the late stance extension torque of the knee as the knee flexes, delivering this negative work from the knee joint as positive work at the ankle to reduce the ankle torque, known as the energy transfer period. This system does not provide net output energy, but rather uses a binary locked/unlocked condition to physically connect the knee and ankle. The combination of these four systems provides energy efficient and natural gait kinematics through the level ground gait cycle with minimal actuation, yet provides opportunity to modify the behavior delivering or removing external energy during the gait cycle and provide different characteristics while attempting unlevel surfaces, sit-to-stand, and stair climbing. The prosthesis was developed using torque and kinematics targets from Winter (2009), using these data to gauge the behavior and requirements of the prosthesis.

#### 2.1.1. Ankle

**Figure 2:**
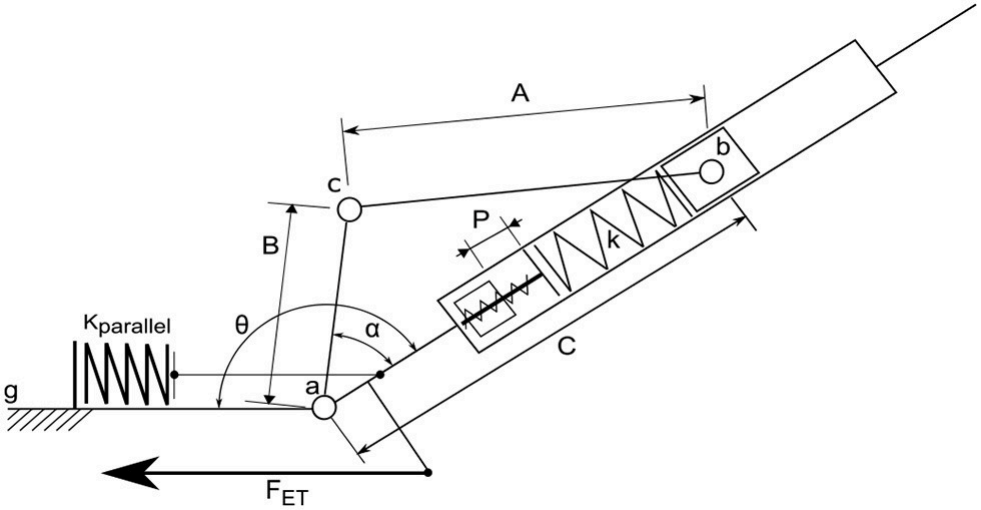
Beta-Prosthesis ankle actuator schematic. Configuration of the selected MACCEPA using rigid linkages. Note the Beta-Prosthesis includes a parallel spring system with a predetermined rest position as well as a manual screw to change the MACCEPA pretension (P).

The ankle can be fully represented by the schematic shown in Figure 2. The total ankle joint (*T*_*A*_, Equation 1) is the torque around joint *a* and is the summation of three torques, the first from the MACCEPA actuator (*T*_*MACCEPA*_), which is dependent on the relative ankle moment arm displacement α and the actuator pretension (*P*), the second from the parallel spring (*T*_*Parallel*_), which is only dependent on the ankle output displacement θ_*A*_, and the third from the Energy Transfer system (*T*_*ET*_), which is dependent on the angles of the knee, θ_*K*_, and ankle and the locking condition *L* (if unlocked *T*_*ET*_ is equal to zero), fully described in section 2.1.2.3.

(1) $$\begin{array}{r} T_{A}=T_{MACCEPA}\left(\alpha ,P\right)+T_{Parallel}\left(\theta_{A}\right)+T_{ET}\left(\theta_{A},\theta_{K},L\right) \end{array}$$

$$\begin{array}{r} \text{where}T_{ET}=\{\begin{array}{cc} T_{ET}\; & \text{if L = Locked}\\ 0\; & \text{if L = Unlocked} \end{array} \end{array}$$

##### 2.1.1.1. Ankle actuator

The ankle of the device is a Mechanically Adjustable Compliance and Controllable Equilibrium Position Actuator (MACCEPA) series elastic architecture (Van Ham et al., 2007; Jimenez-Fabian et al., 2017) with a parallel spring to reduce required peak torques and can allow for smaller motor size. The ankle actuator is composed of a main motive actuator, a series elastic linkage, and a fixed parallel spring, as seen in Figure 2. The actuator torque is created by relative displacement of the moment arm $\bar{ac}$ around the ankle axis *a* from the axis $\bar{ab}$, a displacement called α. This displacement is caused by a motor that is mounted in the shank of the ankle, which in this schematic is represented by the immobile link $\bar{ag}$ to the left. When the moment arm is aligned with the axis $\bar{ab}$, the actuator is in its neutral position and there is no actuator joint torque. In this configuration, the actuator has low stiffness, but as the output is deflected, the natural stiffness quickly rises, much like in a normal ankle. This behavior is fully outlined in Flynn et al. (2015) and Jimenez-Fabian et al. (2015). Notably in the Beta-Prosthesis the main MACCEPA spring pretension (P) is not motor controlled but is simply a manual screw mechanism.

##### 2.1.1.2. Ankle parallel spring

A parallel spring system was added to the ankle to reduce the energy consumption by reducing the necessary holding torque required by the motor and increase the velocity of ankle actuation, as shown in Figure 2. Here the parallel spring engagement depends only on the ankle angle θ, which can be changed by changing the rest position of the parallel spring with shims. This has been done in previous designs, most notably the powered prosthetic ankles from Au and Herr (2008) and Vanderbilt (Lawson et al., 2014).

**Figure 3:**
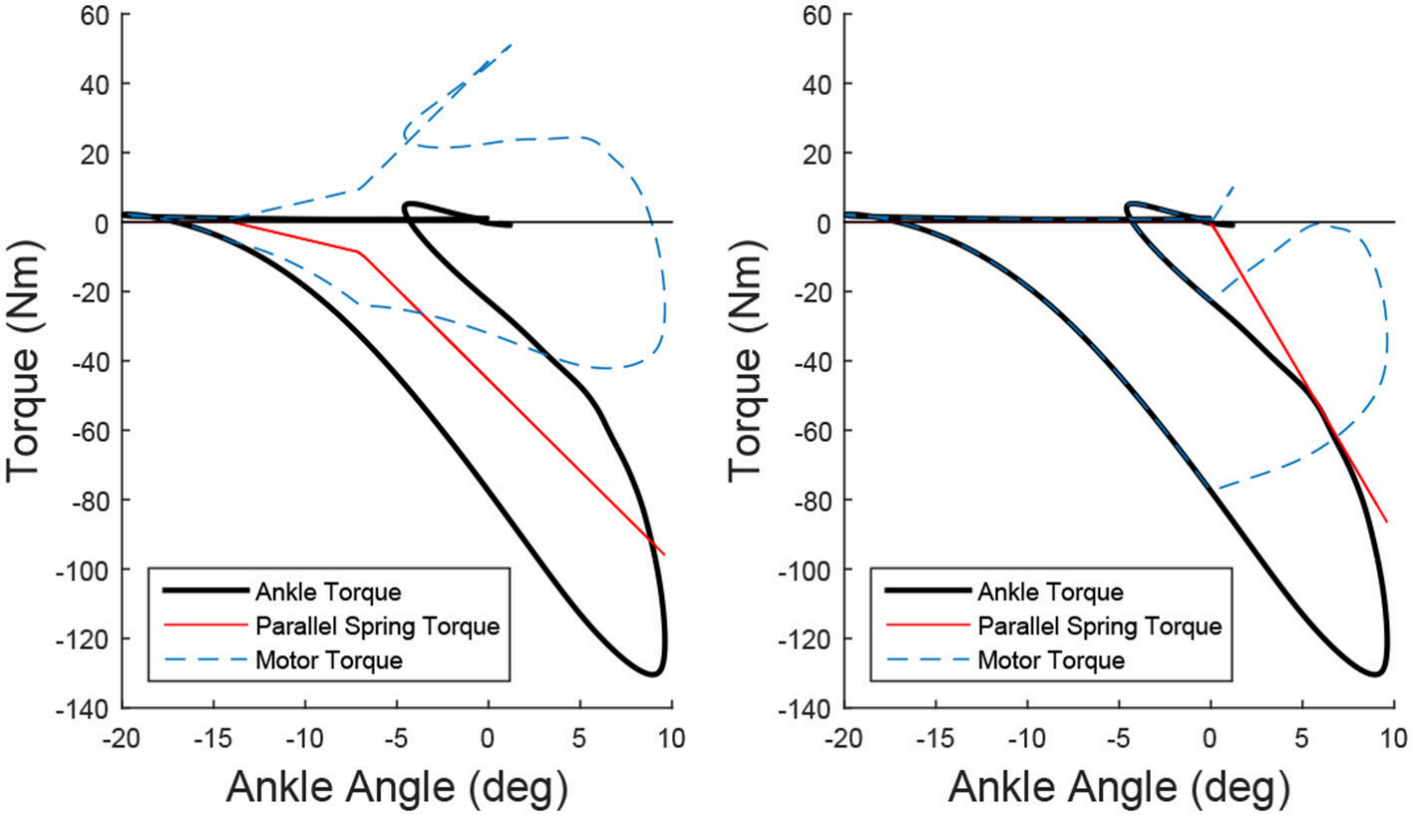
Two examples of adding a parallel spring to modify the Torque/Angle Characteristics of the ankle. By subtracting the torque from the parallel spring (red) from the required ankle torque (black), the required motor torque is determined. In the left example, which was chosen to minimize the peak torque using two linear springs, the peak torque of the actuator is reduced from 130 to 50 Nm. In the right example, the spring was chosen to assist as much as possible without the motor needing to work against the parallel spring during the gait cycle. The peak torque is reduced to 80 Nm.

An example of how the torque output of the actuator is affected by two different parallel spring configurations is shown in Figure 3. In the left of the Figure, a two stage spring, which can be seen as a change in stiffness at –7°, was chosen to minimize the maximum torque while remaining easy to implement with a nested two spring system. The right of the Figure implements a single spring to simply capture the vault over energy of the stance phase, while avoiding loading during the other parts of the gait cycle. This second configuration is a bit more realistic to use, as the motor should never need to load the spring during normal walking, but the reduction of the peak torque is smaller. In addition it only uses one spring, and for tasks where the ankle is passive, such as sitting in a chair, the parallel spring doesn't hinder motion as much. Adding this passive element to the ankle joint does not change the net amount of output work the motor should provide; the integrals of the absolute values of the curves for normal walking with and without the parallel spring remain the same. However, the required peak torque, which is directly related to the current of the motor, in the left example in Figure 3 is greatly reduced from 120 Nm to about 50 Nm for a healthy person of 80 kg, and the right example the peak torque is reduced to about 80 Nm. This reduces the holding torque the motor needs to provide, which is energy lost without providing any output work, but does have an effect on the required power output profile. Overall it allows a reduction in gear ratio of the drive leading to increased actuator velocities and reduction in the electrical consumption of the system.

##### 2.1.1.3. Ankle realization

The left side of Figure 1 shows a CAD model of the Beta-Prosthesis where important features are labeled and can be compared to Figure 2. The parallel spring system can be found in the heel of the device which provides approximately 4 Nm/deg plantarflexion torque. In this design the motor has been placed in the center of the ankle, allowing the motor to be housed within the structure of the shank. The knee system clamps onto this shaft allowing adjustment of the distance and transverse rotation between the knee and ankle axes.

#### 2.1.2. Knee Architecture

**Figure 4:**
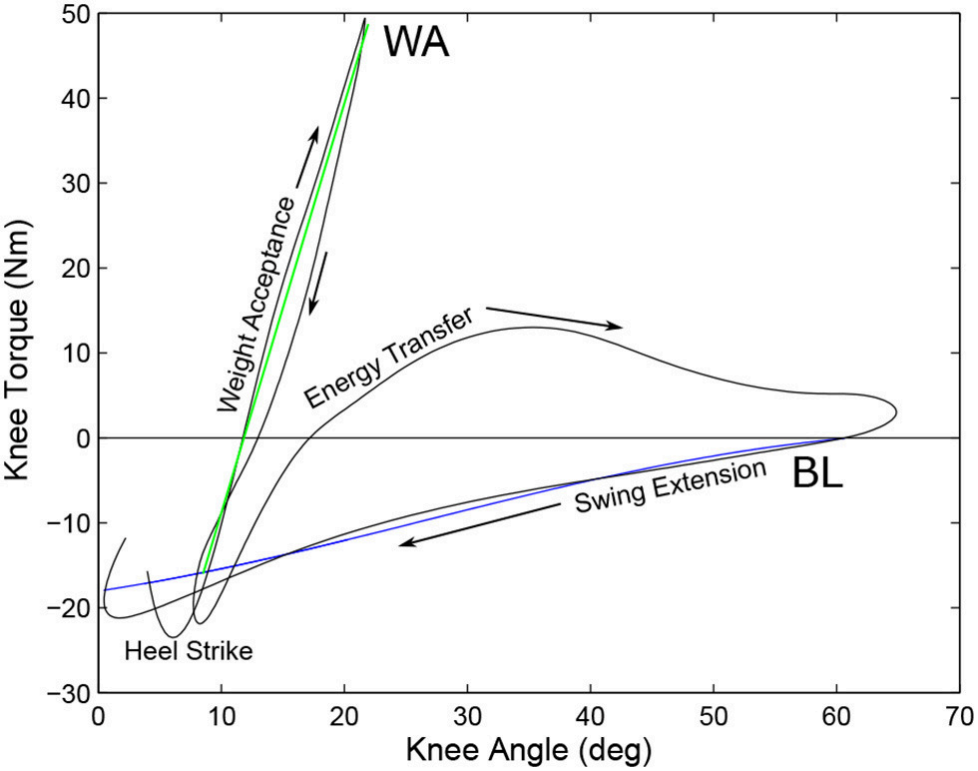
Torque/Angle Characteristics of a 80 kg individual showing the behavior of the Baseline Spring (blue) and the torque during WA (green). The gait cycle begins and ends at the heel strike, progressing to the Weight Acceptance phase where the WA system provides the majority of the torque. After the WA phase, the ET system provides the necessary extension torque to keep the knee from collapsing during pushoff by pulling on the ankle. After full flexion, the ET system is disengaged and the BL spring provides flexion torque to arrest the end of swing phase and is adjusted by the KD system. In this example there is no pretension on the carriage and the carriage is placed in the nominal position (zero torque at 60 degree knee flexion).

**Figure 5:**
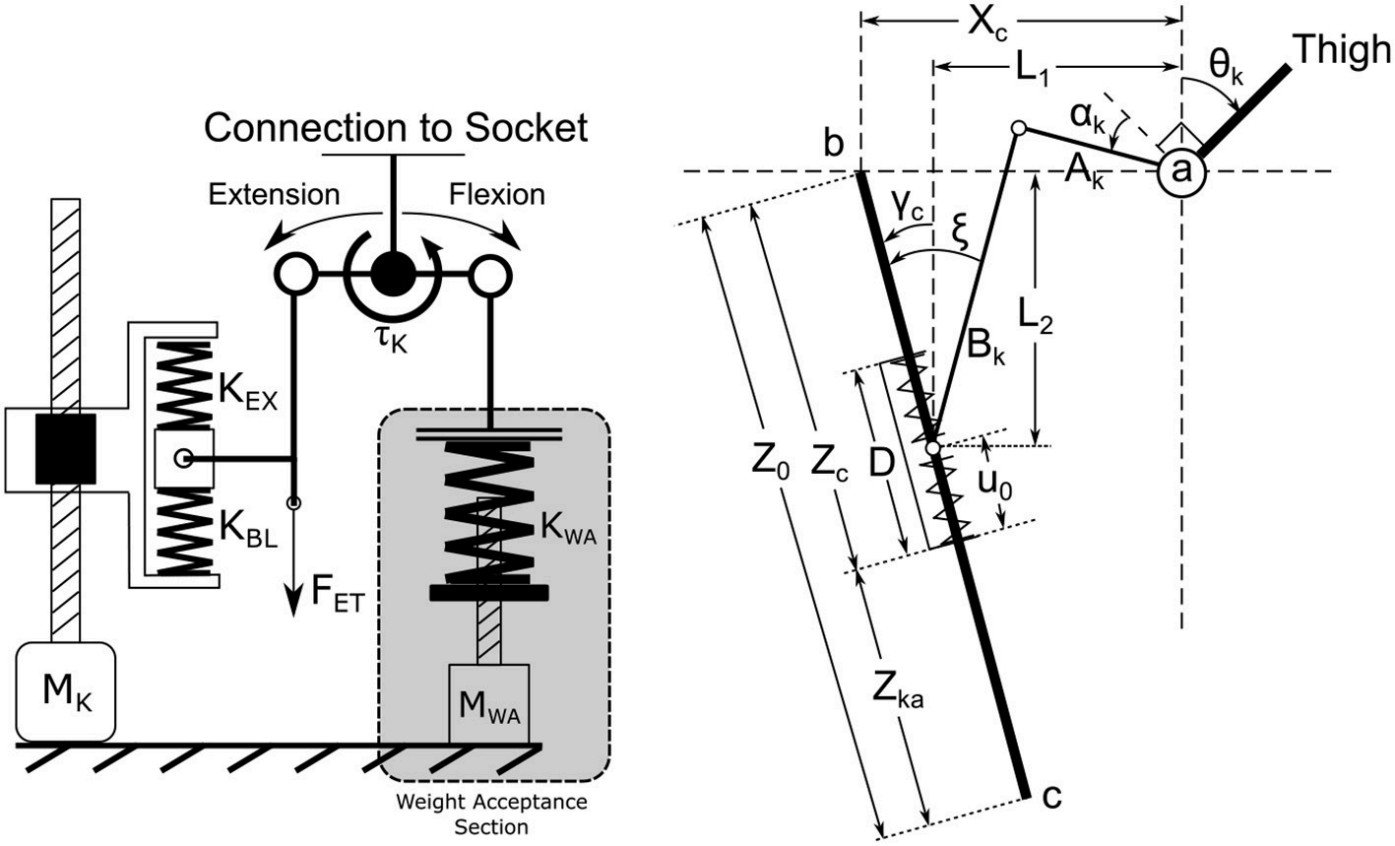
The knee architecture schematic. The left side of the diagram shows the main knee carriage, as well as the baseline (BL) and extension (EX) springs. The BL spring provides the breaking torque during knee extension during normal walking while the EX spring provides the torque during high power extension operations. The carriage moves the rest position of the two springs. This figure also shows the relationship of the WA with the main knee drive. The right figure shows the kinematic definitions used in determining knee actuator torque, as defined in section Knee Actuator Kinematics (see Supplementary Material).

The knee is comprised of three major systems that, when used in combination, can approximate the total knee torque of normal walking with low electrical cost. These systems are the KD, the WA, and the ET system. The roles of each of these systems are outlined in Figure 4. The main KD system consists of a tuned SEA which provides the nominal torque required for normal walking without needing to actuate. When the drive is held at its nominal neutral position (zero torque at 60 degree knee flexion), this drive provides the baseline torque shown in blue in Figure 4. The second system provides a stance flex torque during the weight acceptance phase to reduce collisional costs associated with heel strike, shown in green in the Figure. The third provides torque during the flexion phase of the gait cycle through delivery of negative work from the knee joint as positive work at the ankle, known as the energy transfer period. The physical relationships of these systems can be found in Figure 5 and a general weight and dimension table can be found in section 4.1. This schematic shows the knee motor (*M*_*K*_), the knee Baseline Spring (*K*_*BL*_) and Extension Spring (*K*_*EX*_), and the Weight Acceptance section, which contains the Weight Acceptance motor (*M*_*WA*_) and spring (*K*_*WA*_). The knee joint torque is shown as τ_*K*_ and the force transmitted to the ankle through the energy transfer mechanism is represented by *F*_*ET*_.

##### 2.1.2.1. Knee drive (KD) actuator

The front of the knee houses the KD actuator, as in Figure 1. This actuator consists of a small 50 W motor (Maxon ECi-40) connected through a 5.8:1 gearbox to a 2 mm lead ball screw drive. This actuator can run at a linear velocity of 80 mm/s using a 36 V supply, meaning running from full flexion to full extension in 0.5*s*. This drive is connected to a carriage that houses the series elastic springs, similar in function to the designs of Pratt et al. (2002). The springs in turn actuate on a push/pull rod which drives the knee joint. The knee joint is connected to a standard socket pyramid for interfacing to the subject.

There are two series elastic springs held within the carriage. The Baseline Spring (BL) provides the flexion torque of the knee that is shown in Blue in the Figure when the knee carriage is held at a constant position, corresponding the neutral position at approximately 60 deg. The torque created by this spring, approximately 0.3 Nm/deg flexion, can be modified while under load during all phases of the gait cycle. It is of note that this is much softer than the estimated physiological stiffness seen in Pfeifer et al. (2014) which ranges from 5 to 17 Nm/deg. It is also important to note that energy from the knee motor can be used directly or stored in the knee SEA, even when the WA mechanism is engaged. Opposite to the BL spring is the Knee Extension spring (EX) which provides compliant actuation when stair walking or going from sit to stand. Because the extension moment is theoretically not used during normal walking and only used during high power, non-repetitive motions such as sit to stand, a shorter and stiffer (approximately 6 Nm/deg extension) spring is used, which is better suited to these tasks, used to insulate against shocks, and provide higher forces before full compression.

The right half of Figure 5 shows the kinematic relationships for determining knee torque around the knee joint *a*. Link *A*_*k*_ is directly tied to the thigh while *B*_*k*_ is the red pushrod in Figure 1. Link $\bar{bc}$ is anchored to the prosthesis, and the carriage, which has a length *D*, is allowed to slide along the shaft. The equations governing the knee torque due to the main knee drive can be found in Heins et al. (2018) and are reproduced in Supplementary Material.

##### 2.1.2.2. Weight acceptance (WA) mechanism

Directly after heel strike is the weight acceptance stage of the gait cycle. This is characterized by a spring-like loading and unloading period of the knee joint, as seen in Figure 4 in green. Creating this torque-angle characteristic using the main motor is electrically costly, and would require the actuator to unload and reload the EX spring to create this torque. Also the loading and unloading characteristics are well suited to be replaced by a static spring. The problem with this is the spring needs to be inserted during the stance flexion stage and removed for the swing phase so it does not interfere with the swing flexion, requiring some sort of locking mechanism.

The WA requires a lock that is capable of providing high forces when compressed, but requires little energy to position the spring under low to zero load. The WA does not need to actively provide joint work above the passive storage and recoil of the spring. Also the WA should not unlock when there is a high force on the spring, because if the spring is loaded, it usually means the user of the prosthesis is loading the knee for stability. One way to satisfy these requirements including infinite locking positions, high locking torque, and low continuous locking power is a friction based, non-backdrivable gearing (Plooij et al., 2015).

**Figure 6:**
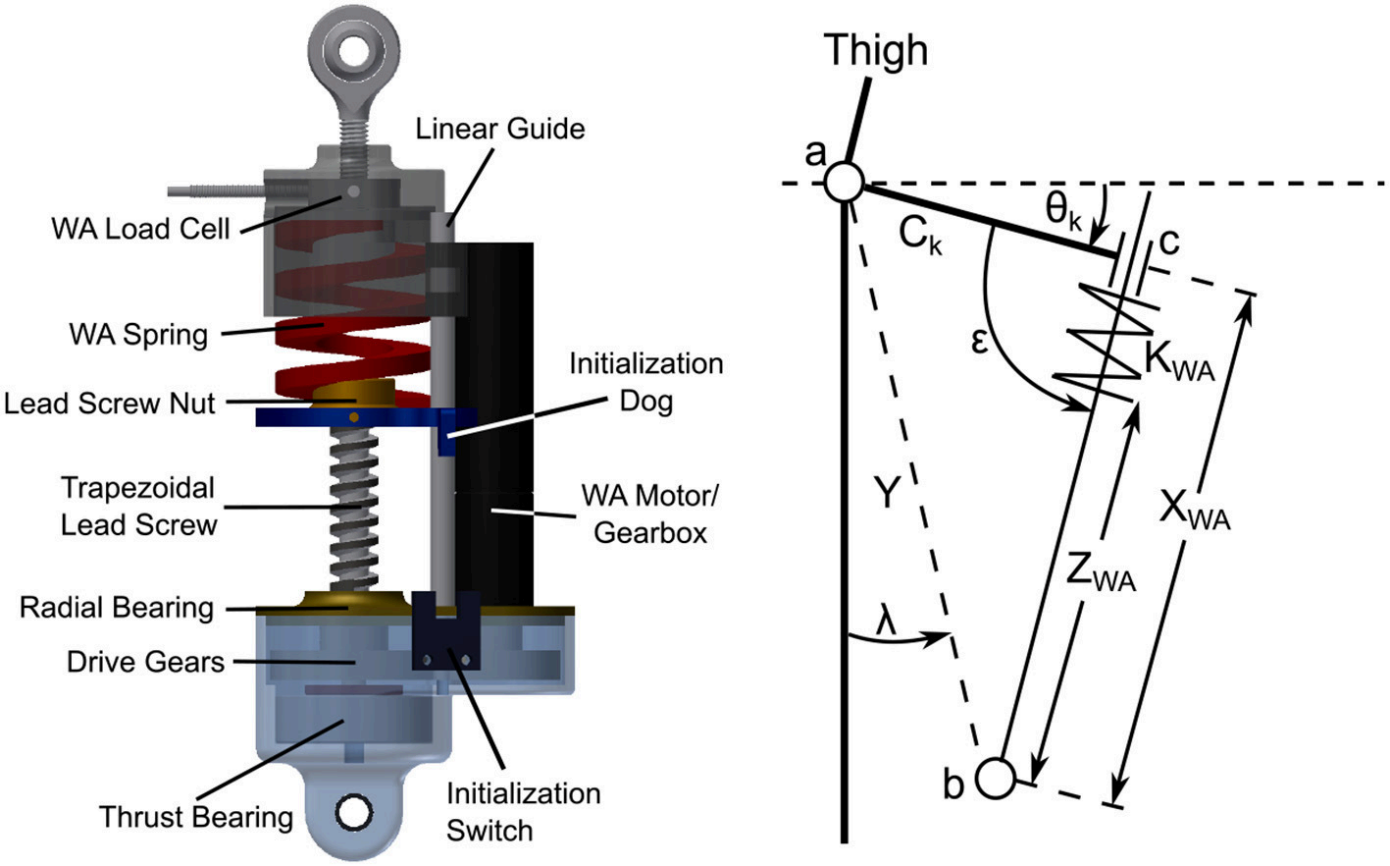
The WA system **(Left)** and a schematic of the system as it is in the prosthesis. The screw drives the spring up and down so the knee interacts with it at a desired angle. The small motor only needs to overcome the friction in the gear drive and nut to move the spring. Initialization is handled by a small optical switch. The WA system schematic **(Right)** shows the relevant relationships needed to calculate the resulting torque from the WA system. The governing equations are presented in section Weight Acceptance Kinematics (see Supplementary Material).

A non-backdrivable screw was chosen as the main drive mechanism, where the rest position of the spring is modified by a small 30 W motor (Maxon EC16) through a gear drive so that as the knee flexes it compresses the spring at a desired angle, as shown in Figure 6. Because the screw is a non-backdrivable trapazoidal screw with a 3 mm lead, the spring is locked in place while under load without the need for motor input. The stiffness of the spring is chosen, when combined with *K*_*BL*_, to provide the torque characteristic shown in Figure 4, represented by the green line. The WA provides a relatively consistent joint stiffness during the stance phase, which corresponds to biomechanical data (Shamaei et al., 2013). Positioning of the WA spring requires a small motor to overcome the friction and inertia of the spring system. The small motor allows the WA to actuate with a linear velocity of 37 mm/s when using a 36 V supply, meaning the device can move from fully unlocked to fully locked in about 0.5*s*. The WA system has been designed with a unilateral constraint so that during swing phase the leg swing can lead the position of the spring. This means if the motor is too slow to track the swing phase, the passive swing extension is not hindered by the WA motor.

This mechanism is attached behind the knee by a moment arm of length *C*_*k*_, as seen in the right schematic of Figure 6. In this schematic the spring is positioned by changing *Z*_*WA*_ by driving the screw. *Y* is the distance between points *a*, the knee joint center, and *b*, the point the WA is anchored to the prosthesis. The slider at point *c* is allowed to compress the spring (*K*_*WA*_) as the knee angle, θ_*k*_, changes. This changes the effective length *X*_*WA*_ and creates the knee torque. Note that if *Z*_*WA*_ is small, the knee will never compress the spring at any angle θ_*k*_. The equations governing this system can be found in Supplementary Material.

##### 2.1.2.3. Energy transfer mechanism

During normal walking the knee primarily acts as a dissipative system, which is one reason why active damping systems can provide relatively good behavior at the knee joint. This necessity of removing energy from the joint provides the opportunity to capture this energy to return to the system at another point of time in the gait cycle. This has been previously done in systems such as the passive WalkMECH (Unal et al., 2013), which uses a spring, push/pullrod, and locking system to deliver energy to the ankle. Systems such as the active MIT CSEA knee simply push regenerated electrical energy from the backdriven drive motor onto the power bus during times of negative work (Rouse et al., 2014).

**Figure 7:**
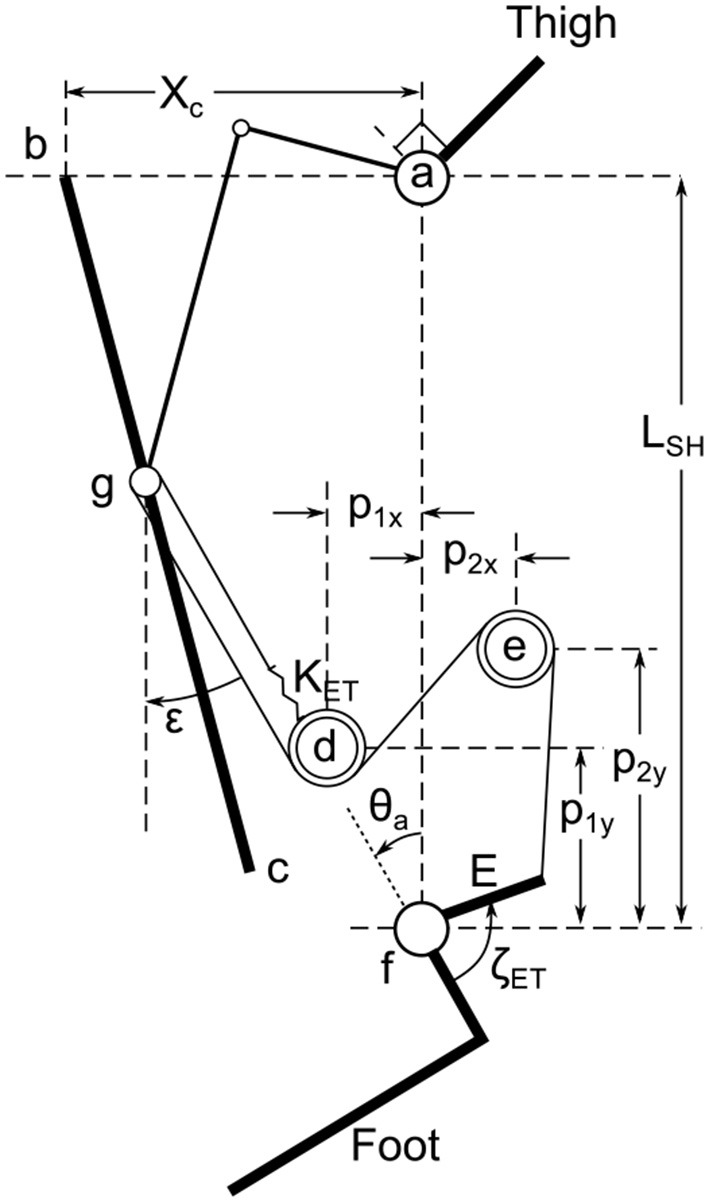
The ET system schematic showing the direct connection of the ankle to the knee joint. Here the knee joint *a* is connected through the moment arm and push rod to the center of the carriage at *g*, constrained to slide on axis $\bar{bc}$. A spring *K*_*ET*_ is connected to the prosthesis at pulley *d* and connected to a cable that wraps around a pulley at *g* and back to pulleys *d* and *e*. The cable then attaches to the ankle at moment arm *E* which when pulled creates a torque around the ankle joint *f*. The positions of pulleys d and e are defined by *p*_1_ and *p*_2_, of which *P*_2y_ can be adjusted through actuation. The distance between the knee and ankle joints is *L*_*sh*_. The governing equations can be found in the Energy Transfer Kinematics (see Supplementary Material).

The ET system of the Beta-Prosthesis intends to capture two major sources of negative work of the gait cycle, work done to stop the lower leg swinging forward at the end of swing phase and negative work done at the knee during late stance that is required to keep the knee from collapsing under the individual while beginning flexion for the swing phase. To do this, there is a cable that directly connects the ankle to the knee when it is locked, and allows the cable to go slack when it is unlocked. A schematic of this system can be found in Figure 7, showing how the ET cable starts anchored to the prosthesis at pulley *d*, wraps around the end of the knee pushrod at pulley *g*, then continues back to pulley *d* and *e* to attach to the back of the foot through moment arm E. Pulley *e* is used as a locking mechanism which is accomplished through the use of a non-backdrivable trapezoidal screw of 3 mm lead and 30 W (Maxon EC16) motor, that was placed on the back of the shank. The screw was used to position pulley *e*, increasing and decreasing the length the ET cable needed to be routed. A stiff spring, *K*_*ET*_ was placed in the cable so the cable force would be limited by the spring displacement. The routing provides a ratio of approximately 2:1 for knee motion to ankle motion. Because the joints are directly connected in tension, a flexion torque of the knee creates a plantarflexion torque of the ankle, but an extension of the knee does not cause a dorsiflexion torque of the ankle. These relationships are governed by the equations in section Energy Transfer Kinematics.

#### 2.1.3. Electrical System

The Beta-Prosthesis was designed to integrate with the CYBERLEGs control and orthosis module systems and therefore does not have onboard control systems or power. The CYBERLEGs control and power system is a completely autonomous system consisting of a wearable backpack housing the control unit and batteries for power (Giovacchini et al., 2015). The control unit is a National Instruments sbRIO-9632 embedded single board computer with an integrated FPGA that is intended to run the prosthesis, the wearable sensory apparatus (Ambrozic et al., 2014), a pelvis orthosis, and high level control algorithms (Ronsse et al., 2013; Ruiz Garate et al., 2017) developed within the consortium.

The sbRIO computer is connected to the prosthesis through a tether cable that carries all of the control signals from the purpose made driver board mounted on the prosthesis. This driver board uses four Maxon 50/5 module driver boards, one for each of the motors in the prosthesis. The tether also transmits the 36 V supply voltage to be compatible with the other components of the CYBERLEGs system.

### 2.2. Bench Testing Setup

**Figure 8:**
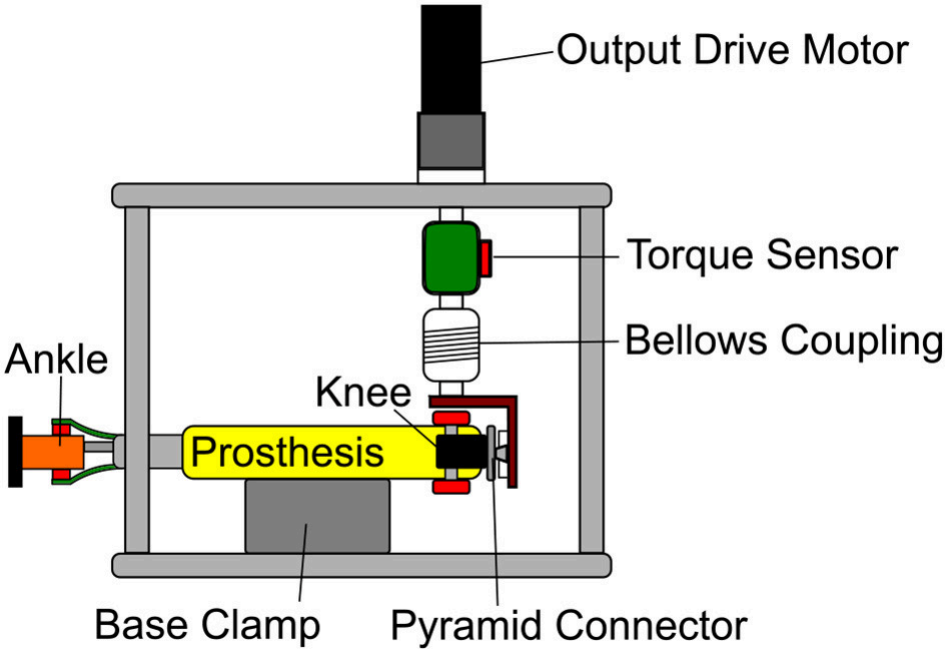
The prosthesis was clamped in a rigid frame with a motor connected to the output of the joint to be tested. This figure shows the prosthesis as it would be positioned in knee actuator or ET system tests. The frame has a large DC motor attached to the prosthesis through a torque sensor, so that the output kinematics of the joint can be varied at the same time as the prosthesis is actuated.

The knee and ankle systems were tested on a custom designed test bench to verify that the system could produce the desired torques and the passive behavior of the knee creates a good approximation of the desired knee torque, as seen in Figure 8. The test bench allows a motor to drive the output of the prosthesis while controlling the relevant joint characteristics, either position of the moment arm or position of the carriage depending on the joint to be tested. The load motor used on the test bench was a Maxon RE 50 with a GP 62 A gearbox driven by a Maxon EPOS2 70/10 motor controller. This motor was connected to the output of the joint to be tested using a rotary torque transducer (ETH Messtechnik DRBK-200-N). The signals for driving the motor and recording the signals were recorded at 100 Hz, with 16 bit accuracy through the sbRIO to guarantee synchronous data collection.

### 2.3. Prosthesis Control System for Walking Experiments

#### 2.3.1. Low Level Control

Low level control of the system of the prosthesis is handled by the ESCON drivers, running a tuned current and velocity feedback loop. The velocity signals used by the ESCON are generated by the FPGA of the sbRIO running a control loop at 1,000 Hz. Position commands are calculated by the real time system at 100 Hz and fed to the FPGA. Position commands that are tracked by the real time system are calculated by the Top Level Control.

#### 2.3.2. Top Level Control

**Figure 9:**
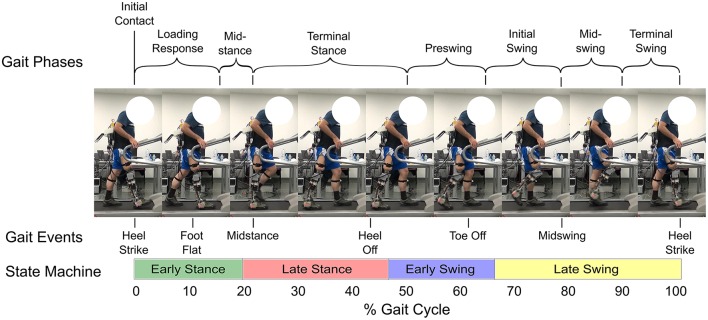
Normal level ground gait cycle (adapted from Cuccurullo, 2004). The figure shows how the different states correspond to the normal gait cycle, with the shaded leg representing the prosthesis. Also displayed are frames from a video of an experiment showing the gait cycle of the amputee. Note that these transition points are not fixed with respect to the gait cycle percentage, but rather to certain measurements made by the WSA (see Figure 10) and therefore can change. Written informed consent was obtained from the subject for the publication of this image.

**Figure 10:**
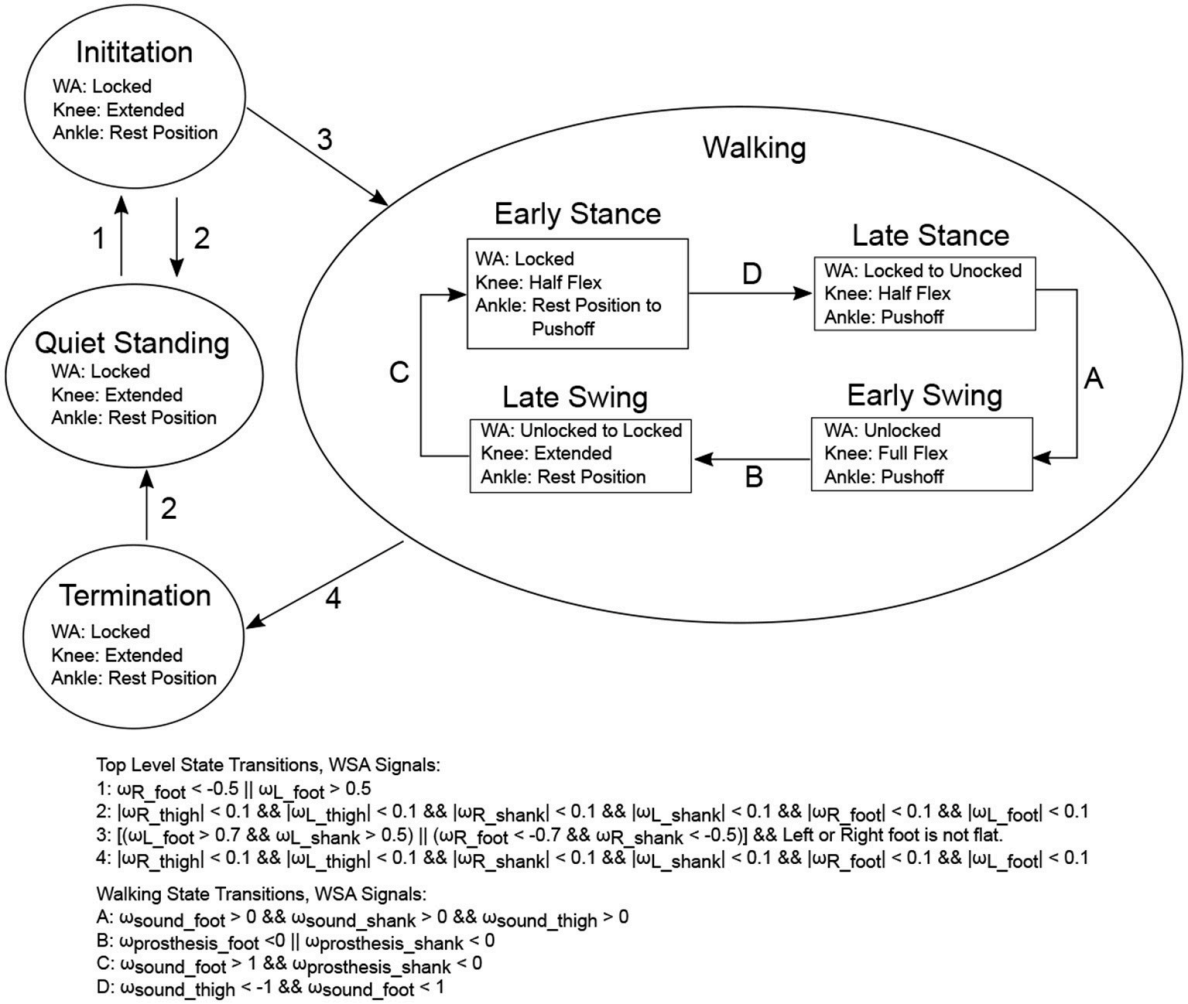
The CYBERLEGs Walking State Machine. The CYBERLEGs WSA was used to create the triggers for state transitions, using the angular velocity, ω(*rad*/*sec*), of the different limb sectors. The pressure insoles were used to determine if the feet were on the ground. Top level systems are shown in circles, and the positions of each of the knee, ankle, and WA are shown. The exact values for each of these setpoints was determined by empirical trials.

Control methods for the Beta-Prosthesis utilized a modified Intention Detection (ID) system and Wearable Sensory Apparatus (WSA) controller with a finite state machine as discussed in Ambrozic et al. (2014) and Parri et al. (2017). Here the state machine has been modified to include the new WA mechanism positions as well as the KD command positions. The state machine of the WSA contained a number of levels, the first including a quiet standing, gait initiation, and gait termination phases. From the gait initiation phase the main walking state machine was entered. The walking state was broken into four different sub-states named, from the point of view of the prosthesis, the Early Stance (State 1), Late Stance (State 2), Swing (State 3), and Late Swing (State 4) phases of the gait cycle as seen in Figure 9. These states were triggered by a combination of the WSA angular velocity sensors as well as signals from pressure insoles. Each of these states was designed to capture a specific gait event, heel strike, heel off, toe off, or terminal swing phase. The state machine system along with state transitions is summarized in Figure 10.

It should also be stated that these position setpoints (locked, unlocked, half flex, etc.) were tuned for the individual's self selected speed and were used only for level ground walking.

### 2.4. Subjects

The male subjects (*n* = 4) had an age range of 63 (*SD* = 11) years, a weight range of 61.8 (*SD* = 2.63) kg, and a height range of 173.75 (*SD* = 5.06) cm. Three had amputations due to traumatic injury and one was a dysvascular patient with an average of 11 (*SD* = 10.67) years since their amputation. Their mobility levels varied from were K1 (the dysvascular subject), to K3 (two subjects), as defined by the Medicare Functional Classification Level.

## 3. Experiments

### 3.1. Ankle Bench Testing

**Figure 11:**
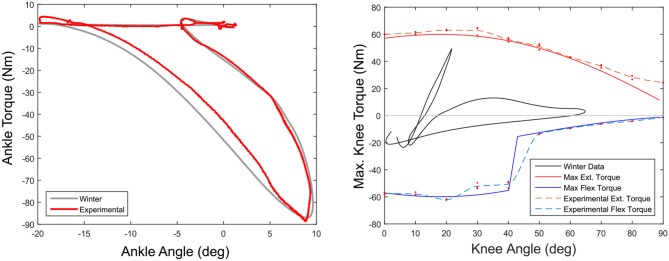
Ankle actuator torque using moment arm control **(Left)**. The moment arm tracks a position trajectory to create the output torque (red) creating the Winter Target output torque (gray). The test was run to a peak of only 90 Nm because of limitations in the bench test motor. The maximum flexion (blue) and extension (red) torques for the knee actuator are shown in the **(Right)** figure. This does not include the torques created by the WA spring mechanism.

The ankle was placed in the test bench described in section 2.2 with the ankle output connected to the torque transducer. The output motor was used to drive the ankle angle along the Winter trajectory at 1.5 s/stride while the ankle moment arm was commanded to provide the ankle torque for the given time in the gait cycle. Although the ankle is capable of providing 130 Nm of torque during the gait cycle, the test bench motor was only capable of driving the output to 90 Nm due to limitations in the current the external motor driver was able to provide. Therefore a Winter torque/angle trajectory was selected with a peak torque of around 90 Nm. Results can be found in Figure 11 (Left).

### 3.2. Knee Actuator Bench Testing

In this experiment the knee was locked at a fixed angle and connected to the test bench torque transducer. The carriage was commanded to deliver the maximum joint torque. This was repeated three times for both flexion and extension every 10° of knee flexion, from full extension to full flexion. The actual joint torque was recorded and maximum measured torque was compared to estimated maximum torque. Results can be found in Figure 11 (Right).

### 3.3. Knee With ET Bench Testing

The prosthesis was placed in the test bench setup with the output motor of the test bench connected to the knee joint. The ET system was then commanded to lock and unlock from approximately 43% to 64% of the gait cycle, corresponding to the time of negative work of the knee joint. The ET cable tension was measured with a load cell while the knee and the ankle were commanded to track respective position trajectories. The ankle motor was commanded in output position mode, pinning the kinematics of the ankle to the Winter trajectory regardless of the torque required.

### 3.4. Preliminary Walking Experiments

As part of the preliminary validation of the prosthesis system, four amputees were subject to a 3 min treadmill experiment. Each subject attached the prosthesis to their existing socket through a standard prosthesis pyramid adapter and a visual alignment was performed by a prosthesis technician. After a short session of approximately 15 min for tuning of the prosthesis state machine parameters from section 2.3.2 over a level ground 10 meter long catwalk, the subject was asked to walk at a self selected pace on the treadmill for 3 min to investigate the behavior of the knee joint. This self selected speed was determined by slowly increasing the speed of the treadmill in 0.2 km/h increments until the subject felt it was too fast to sustain. The speed was then reduced until the subject was comfortable. Subjects were asked to minimize the use of the handrails, although to not remove their hands from the rails in case of an emergency. Subjects for the experiments were selected and approved by the Ethical Committee of the Don Gnocchi Foundation (FDG), Florence Italy, and experiments were conducted under FDG supervision. Data was recorded from the prosthesis during the treadmill testing period.

During the validation with subjects, the ET system was not used for testing. This was because the control of the ET system would not allow for kinematics that varied greatly from the desired Winter kinematics, leading to high forces as well as ineffective gait. This was mitigated though the use of a modified KD trajectory, allowing the subjects to walk. This is discussed in greater detail in section 5.2.1.

## 4. Results

### 4.1. Design Results

A list of prosthesis design values and prosthesis characteristics are found in Table 1. These values were used in simulation and during the testing session.

**Table 1:** Selected prosthesis characteristics used in simulation and for final design.

**Property**	**Value**	**Units**
Active DOF	2	knee, ankle
System Voltage	36	VDC
Ankle Moment Arm Length (*B*)	50	mm
Ankle Linkage Length (*A*)	50	mm
Ankle Actuator Spring Constant (*k*)	130	N/mm
Ankle Actuator Spring Weight	113.4	g
Ankle Parallel Spring Constant	94.20	N/mm
Ankle Parallel Spring Weight	60	g
Ankle Gear Ratio	320:1	-
Ankle Winding Voltage	48	V
Ankle Max Torque	130 (@15A)	Nm
Ankle Continuous Torque (no parallel spring)	30.6	Nm
Ankle Range of Motion	–30 to 20	deg
Shoe Size	42	EU
Knee Gear Ratio	5.8:1	-
Knee Ball Screw Ratio	2	mm/turn
Knee Max Torque	~70	Nm
Knee Continuous Torque	~55	Nm
Knee Range of Motion	0 to 95	deg
Baseline Spring Constant (*K*_*BL*_)	10.7	N/mm (each spring)
Baseline Spring Length	64	mm
Baseline Spring Diameter	16	mm
Baseline Spring Mass	19.7	g
Extension Spring Constant (*K*_*EX*_)	89.1	N/mm (each spring)
Extension Spring Length	32	mm
Extension Spring Diameter	16	mm
Extension Spring Mass	15.6	g
Weight Acceptance Spring Constant	300	N/mm
Weight Acceptance Spring Rest Length	38	mm
Weight Acceptance Spring Diameter	32	mm
Weight Acceptance Spring Weight	90	g
Prosthesis Overall Mass	~5	kg
- Knee Module Mass	1,736	g
- WA Module Mass	440	g
- Ankle Module Mass	1,850	g
- Electronics	300	g
Prosthesis Height (Overall, longest)	500	mm
Knee to Ankle Length (Shortest)	355	mm
Knee to Ankle Length (Longest)	395	mm
Knee to Pyramid Top Length	30	mm

### 4.2. Bench Testing

#### 4.2.1. Ankle Torque/Angle Testing

The results of the ankle torque/angle characteristics from the bench testing device can be found in Figure 11. The actuator provides a torque output that is well with the standard deviation of the Winter ankle torque/angle behavior.

#### 4.2.2. Knee Actuator Torque Testing

Figure 11 (Right) shows the estimated maximum torque (solid blue and red lines) and experimental values (dotted blue and red lines) achieved by the actuator and compared to the normal torques during walking (black).

#### 4.2.3. Knee With Energy Transfer

**Figure 12:**
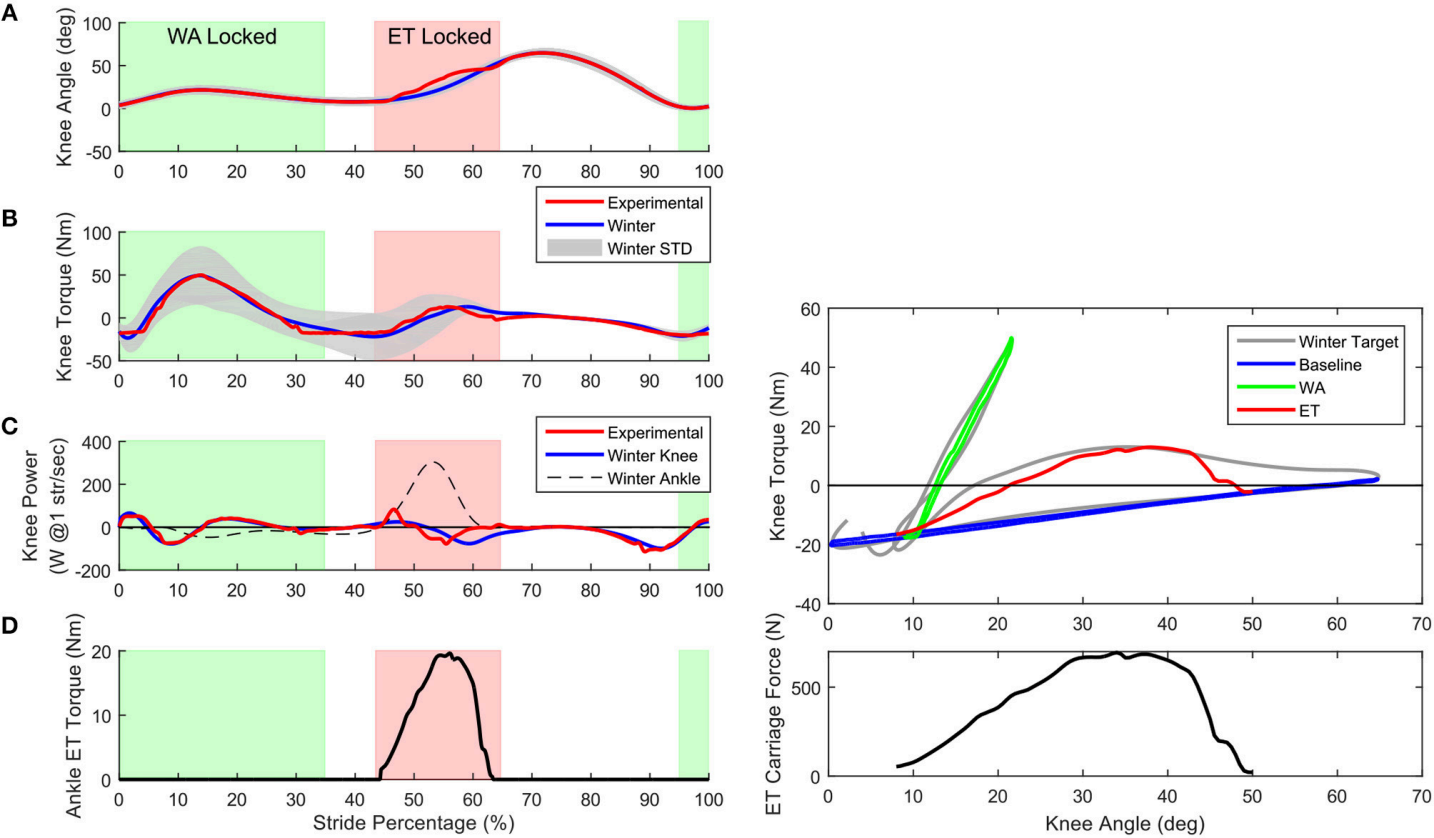
Bench test results of the prosthesis with ET system. **(A)** The knee angle of the prosthesis (red) while using the ET in the current configuration. Note that while the ET is locked, the knee angle deviates slightly from normal Winter angles (blue). **(B)** The time dependent behavior of the torque shows the knee torque well within the standard deviation of the Winter data for almost all of the gait cycle. Using just the passive systems of the prosthesis can provide a good approximation of normal knee torque. **(C)** The knee power (red) shows the shift in the power peak to better align with the peak in the ankle power (dashed black). By bending the knee earlier, the power of the knee is better suited to transfer to the ankle during the maximum pushoff. **(D)** Shows the torque of the ankle provided only by the ET system. This torque would replace ankle motor torque, reducing electrical consumption. Right Top graph: Passive behavior of the knee actuator including ET. Using only the BL (blue), WA (green), and ET (red) provides the knee with a good approximation of the normal Winter knee torque. The knee actuator carriage is kept constant. The green line is the output torque of the combination of the BL and WA while the WA is locked, the red line is the combination of the BL and ET. Right Bottom: The force in ET cable during the time the ET is engaged. The force in the cable peaks at around 700 N, which is applied at the ankle, reducing the ankle motor torque.

Figure 12 shows the results of the bench tests of the prosthesis while using the energy transfer system. The ET system was able to transfer approximately 8.2 J/stride from the knee to the ankle during this trial.

### 4.3. Preliminary Walking Experiments

**Figure 13:**
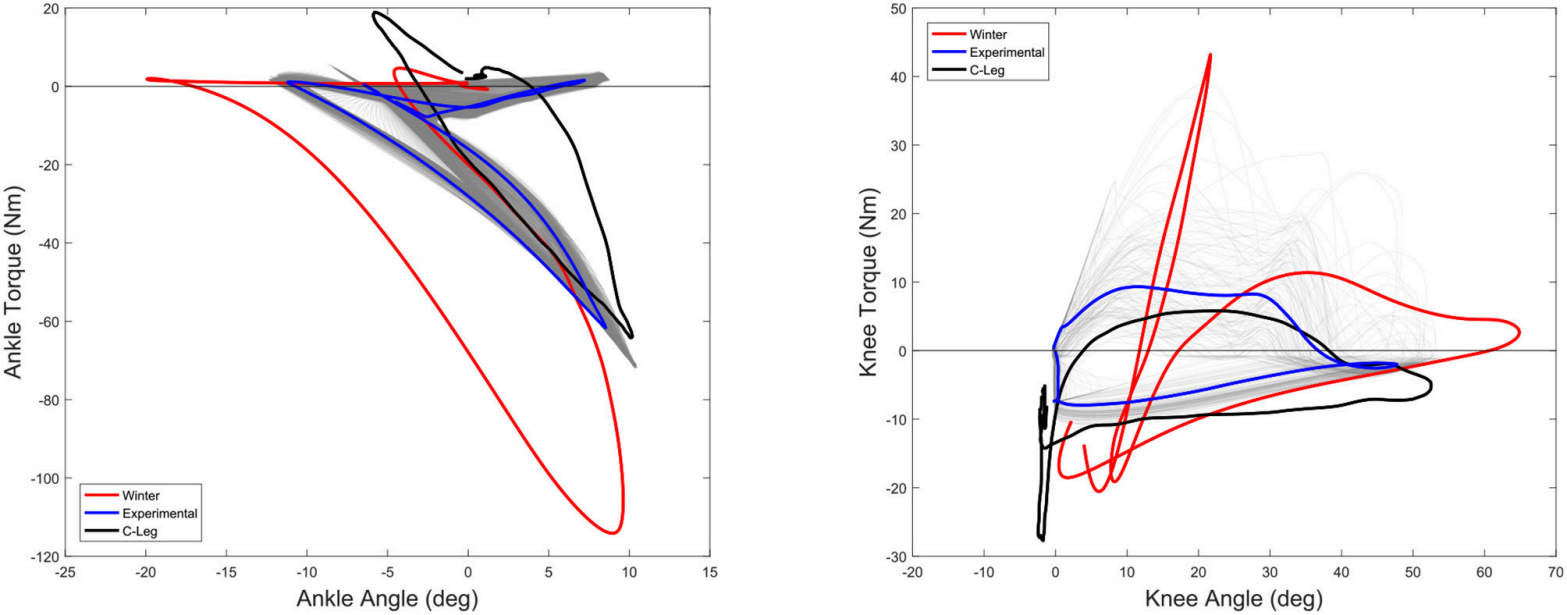
The experimental mean torque-angle characteristics of the ankle **(left)** and knee **(right)** during amputee experiments (Blue) at 2.2 km/h compared to the Winter reference (4.8 km/h) (Red). All 126 strides of a representative subject are shown in gray traces, illustrating the variation between steps. Also shown are data from a widely prescribed C-Leg microcontroller prosthesis (black) (Segal et al., 2006). The desired ankle pushoff torque was much lower than the normal walking at the request of the subjects, in part due to the lower walking speed of the tests. Average energy injection from the ankle was about 2.8 J per stride. The knee behavior of the powered prosthesis ended up providing a microcontroller-like torque angle characteristic, mainly because of the lack of stance flex.

A representative example of a subject walking on the treadmill for 126 strides at a speed of 2.2 km/h and average stride duration of 1.76 s/stride (*SD* = 0.07) are presented. The torque angle relationships for the ankle (left) and knee (right) are found in Figure 13 and shown compared to the typical behavior of a microprocessor controlled C-Leg (Segal et al., 2006). The average energy injection from the ankle was about 2.8 J/stride.

**Figure 14:**
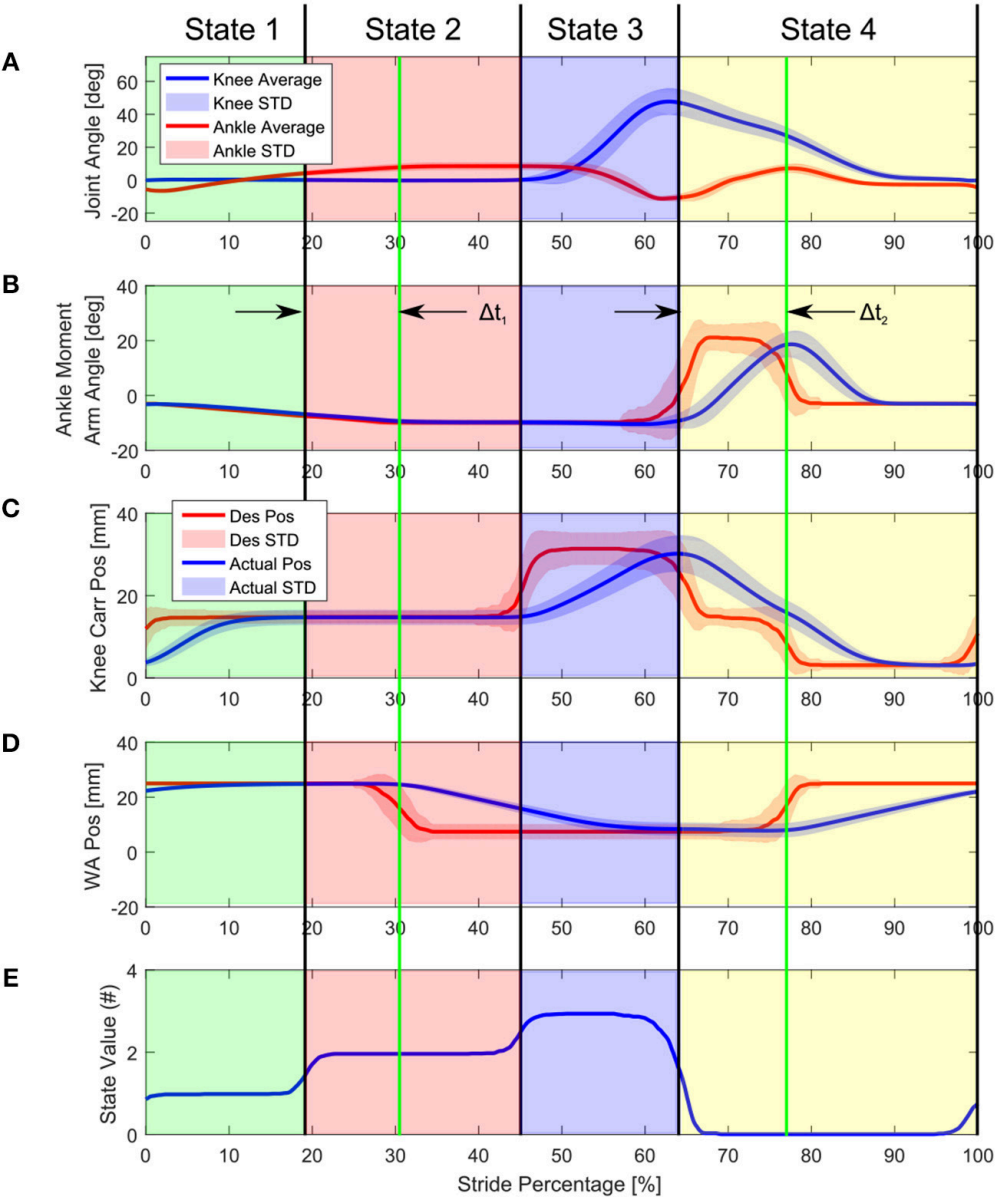
Knee angle, Knee Drive carriage position, and WA position during treadmill walking. The desired position of the WA and KD carriage are shown in red, here the gait state machine determines when the position setpoint should be changed, the position of which was determined experimentally through tests over level ground. The state changes do not happen at exactly the same time during the gait cycle on every step because of variances in the signals given to the WSA, resulting in curved averaged state values. Δ*t*_1_ and Δ*t*_2_ are experimentally determined delays between the beginning of State 2 and the unlocking of the WA and between State 4 and the locking of the WA.

Figure 14 shows the average behavior of the actuators and joints during a walking trial on the treadmill as well as the timing of the state machine transitions during the gait cycle. Also shown here are the two absolute time based transitions Δ*t*_1_ and Δ*t*_2_ which were experimentally determined delays between the beginning of State 2 and the unlocking of the WA and between State 4 and the locking of the WA. The state transitions are determined by the WSA.

Figure 14B shows the ankle moment arm desired position and actual position. The ankle was only commanded to –10° of plantarflexion, as opposed to the calculated –20° for full plantar torque. This was as requested by the amputees.

Figure 14C shows the desired and actual knee carriage position. Because the knee does not have the energy transfer mechanism the knee actuator is used to provide flexion and extension torques when necessary. Even though the ET system was not included in the initial trials, the KD system of the prosthesis was able to actively compensate for this missing component, at the expense of a less electrically efficient gait cycle.

Figure 14D shows the WA motor position. At the heel strike beginning of the gait, the WA is in the locked position. During 40–80% of the gait cycle, the knee motor setpoint is set to a flexed position to help the amputee with ground clearance during the initial swing phase. The knee joint flexes to around 50 degrees and the knee carriage position is moved back to extended at the end of the swing phase. The knee WA locks at the end of swing to guarantee a safe heel contact.

Figure 14E shows the state machine state during the gait cycle. The state machine state was determined by the WSA, and therefore did not happen at exactly the same time during the gait cycle on every step, but because the gait was rather consistent, the transitions happen at approximately the same time. This is seen as the sloping averages in the graph. The states roughly correspond to early stance (Value 1), late stance (Value 2), early swing (Value 3), and late swing phases (Value 0) of the prosthesis.

### 4.4. Prosthesis Energy Consumption During Trials

From previous power consumption measurements, the total device consumes about 65 J/stride. Of this, the WA requires about 10 J/stride regardless of the speed of the gait. Electrical model estimations show that during this trial the ankle uses about 19 J/stride while the knee uses about 23 J. These values seem to be considerably less than older versions of the Vanderbilt prosthesis (91 J/stride), although this was at 5.1 km/h and 87 steps/min with a higher joint work output (Sup et al., 2009) and was considerably higher than the CSEA knee (3.6 J/stride) which had a net negative joint work and was able to partially power its own electronics through regeneration (Rouse et al., 2014). These values should be taken only as general guidelines due to the significantly different testing conditions and subject behaviors in each study.

## 5. Discussion

Testing of the device was divided into two different sections, bench testing and the patient trials. Bench testing of the device shows the device can work as designed when the external kinematics are imposed, with good ankle and knee torque approximation of the biomechanical data at speeds close to the actual speeds of the tests. Patient trials showed that all four patients were able to walk with the prosthesis using the CYBERLEGs WSA over level ground and on the treadmill with minimal (< 1 h) training. Overall these tests show it is possible to find walking gaits that allow for ambulation with actuators that utilize soft series elastic springs, although the overall behavior was not the same as averaged normal gait as defined by the Winter biological data. An example of the typical gait can be seen in the Video in Supplementary Material which shows that qualitatively smooth gait progression and a small energy injection at the ankle was possible.

From the patient experiments, it was apparent that ET design and control was insufficient for gaits greatly differing from the average kinematics, leading to the loss of ET function and in some cases interfering with the gait cycle. Due to this loss of ET function, the knee actuator control was highly modified compared to the original design. This resulted in gait that was closer in behavior to a passive knee than the average biomechanical data. This was exacerbated by short training periods that were insufficient to allow the needed level of familiarity with a device that operates dramatically different from their currently prescribed prostheses.

### 5.1. Bench Testing

Even though the tests were slightly limited in speed and magnitude compared to the original torque and angle targets, we have been able to show that the power consumption of the knee actuator alone (i.e., without the ET system) does reduce the energy consumption of the motors compared to a direct drive system (Geeroms et al., 2017, 2018). This is due to the division of the torque angle characteristics of the knee into different sections, utilizing the WA system for the high stiffness required after heel strike and energy storage of the BL spring during stance and swing phases. Ankle behavior also was able to utilize the energy capture and return of the ankle SEA and parallel springs. Bench testing with the ET system also shows that it is possible to transfer energy (at least 8.2 J/stride) from the knee to the ankle, at least under specific kinematic and loading conditions.

#### 5.1.1. Ankle Bench Testing

The ankle has a bit of hysteresis primarily coming from friction in the parallel spring mechanism, which can be seen in Figure 11. Overall the ankle was able to reliably reproduce the torque angle characteristics of the normal Winter data.

#### 5.1.2. Knee Bench Testing

The right side of Figure 12 shows predicted and actual knee actuator torque measurements while bench testing the knee actuator. The estimated torque values come from a maximum 2000 N axial force on the actuator carriage. To reach this high torque the spring must be fully compressed for both the extension and flexion torques. The low torque region of the flexion torque is where the carriage does not have enough displacement to fully compress the spring. The actual joint torques of the knee are well estimated by the predictions.

#### 5.1.3. ET Bench Testing

Using the force in the cable and the moment arm of the ET on the ankle, the torque on the ankle can be computed. Multiplying this with the displacement of the ankle gives the energy transferred from the knee to the ankle. In this example the value is 8.2 J, which is about 80% of the available energy from the knee and about half of the required energy for pushoff, a considerable savings if it can be replicated in walking individuals.

The top graph in Figure 12 shows the torque-angle characteristics of the full prosthesis knee including the KD, WA, and ET in the test bench. The contributions of each system is illustrated by a different color in the graph. When the graph is only blue, the BL spring is the only component providing knee torque. When green, the WA and the BL systems combine to create the knee torque. When red, the ET system and the BL combine to create the displayed torque. The bottom graph of the Figure shows the force in the cable of the ET system during activation. This cable pulled on the ankle with a moment arm of approximately 6 cm, resulting in an energy transfer of around 8.2 J.

The knee trajectory was slightly modified to allow the best fit between knee and ankle torques. This also had the effect of shifting the knee power a bit earlier which allowed the energy transfer to align better with the ankle pushoff. In normal walking, the negative work from the knee is slightly after the pushoff of the ankle, as shown in Figure 12C. Also if one wants to track the Winter ankle power exactly, if the ET transfers normally, there is a requirement of the ankle motor to absorb the late energy of the ET system, as discussed in Heins et al. (2018). By shifting it earlier more energy can be transferred without needing to dissipate mistimed energy at the ankle. In normal walking the net work output of the knee is around –14 J, while with our modified kinematics the output work was around –10 J, but the time shifting of the negative work allowed more energy transfer.

### 5.2. Walking Trials

Four subjects were able to walk on level ground after a short training session. Two of the four were able to increase their self selected walking velocity by approximately 0.2 km/h over their own prosthesis. Figure 14 shows the averaged results for the experiment. Figure 14A shows the angle of the knee joint during walking. As the amputees were not used to walking with this prosthesis and did not train for a considerable amount of time, they did not use the stance flex as it was designed to be used and preferred a straight leg during walking. Based on subject feedback during the tuning session, the WA motor was given a sufficiently high setpoint which effectively locked the knee joint in extension. This effect is seen during the first half of the gait cycle, as there is no knee flexion. Adapting this for different terrain and speeds are a couple of focuses of future study for the state machine controller. Other types of controllers can also be implemented on the device and will be investigated, such as the motor primitives methods that have been developed within the CYBERLEGs consortium (Garate et al., 2016; Ruiz Garate et al., 2017).

The ankle was able to inject energy into the gait cycle, but this amount was small during these tests. The ankle used a very small pushoff angle, at the request of the subjects. On average the ankle performed about 2.8 J of work per step, which is far from the 17 J of the target walking, but better than the slightly negative total work provided by passive devices.

The question then arises if users settle into these ideal torque and angle characteristics if the prosthesis is able to do so on the test bench. From the initial patient trials with the prosthesis, it was clear that the subjects did not have a Winter-like average gait torque/angle progression, with joint torque and angle characteristics varying greatly from the original targets.

#### 5.2.1. Issues With the ET System

The largest deviation from the original design was the inability to utilize the ET system. The ET system was designed with specific required output kinematics to correctly harvest knee negative work and deliver that work to the ankle. Deviations from these kinematics caused a dramatic rise in the cable system past the ET design criteria. For example under normal conditions the ET could expect to see approximately 600 N of tension during walking (Heins et al., 2018). If the kinematics were changed to those of a standard C-Leg walker, these forces could increase to higher than 4000N. The deviant kinematics, in particular the timing between knee and ankle motions, and increased forces prohibited the ET system from unlocking at the correct times. As a result, the prosthesis ankle was not able to dorsiflex at the beginning of the swing phase, increasing the chance of stumbling and resulting in an unsafe situation for the amputee.

Removing the ET modified the behavior of the KD because the original torque angle behavior of the prosthesis depended on this system to provide extension torque during the late stance. This modification of the KD required the actuator to move much further distance than originally intended to provide this extension torque, which limited the peak velocity of the knee.

It is possible that if proper feedback control for the ET mechanism was created, then it might be possible to safely use the system by limiting the tension in the cable, although because the ET was not designed to actuate under load, this was not implemented. In addition if the ET is then a fully active system rather than a clutched system as designed, there must be a controller that can watch the knee and ankle joints and predict periods of time when the knee would dissipate power and the ankle provide kinematics that were suitable to receive this energy, if those periods exist. Another way of solving this issue is to strictly control the output kinematics, which would guarantee the knee and ankle relationships. This method would not necessarily result in a reduction of motor electrical consumption or a suitably stable gait. Modifications to the ET system must be made to further examine this aspect of the design in walking trials.

### 5.3. Control System Modifications

The control system of the prosthesis was designed to change the configuration of the prosthesis in order for the output torque to match some quasi-static torque target assuming that the output kinematics of the system would then converge to the normal gait kinematics. This method makes a of assumptions, most importantly first that if the average joint torques are obtained, the person would naturally have kinematics which are about normal and second that deviations away from normal joint torques caused by external disturbances would be sufficiently handled by the natural impedance of the prosthesis and the control of the person using the prosthesis. The control method was not designed to be a classical impedance or torque based system using output feedback to generate a specific stiffness or trajectory. On the bench this works well because the output kinematics of the system are constrained by the output motors, and therefore the joint torques and kinematics are as expected.

When both the torque and kinematics are unconstrained, the person tends to walk very differently than expected, particularly at the knee. While ankle kinematics and torques were somewhat normal for the low input power that was desired, the knee behavior was far different. To find gait cycles that were capable of safely walking, the ET needed to be disabled. Because of the lack of the ET system during the walking trials, the control of the system was modified so that the KA could replace the functionality of the WA system. This included changing the state machine to follow a different trajectory than that with the ET enabled, ultimately implementing a very simple flexion/extension motion for the knee, as was determined from feedback from the subjects. For each of the 4 states of the gait cycle, the subjects were asked what behavior they would like to have from the prosthesis and the position threshold for the state was determined.

#### 5.3.1. Normal Torque/Angle Characteristics as Target

The Beta-Prosthesis was designed with the intention of providing the normal torque and kinematics of a leg, as determined by average healthy gait. With this particular design and controller there is an implicit assumption that the prosthesis has a similar mass and moment of inertia as an average human leg, because the target torques are dependent on these aspects. This prosthesis has been built with this as a constraint, but tends to lead to a relatively heavy prosthesis and associated problems, such as socket pistoning. From discussions with the subjects during the trials, while walking while powered this extra weight is not noticed until the prosthesis performs poorly or is not actuated, and then the weight is highly detrimental.

It should be noted that even without the normal biological torque/angle joint progression the patients were able to walk at speeds equal to or above those while using their every day prosthesis. So how necessary is it that the prosthesis really track the normal gait characteristics? Indeed some extremely fast transfemoral amputee sprinters find that the design of their passive prostheses may not need a knee joint at all, relying on the prosthesis design to generate the pushoff and using the hips to provide ground clearance.

Volumetric oxygen measurements with almost all current prostheses are generally 10–30 percent higher than normal walking, and 50–100 percent higher at maximal speeds of walking (Genin et al., 2008). Many have suggested this is because of dealing with gait asymmetry, and energy consumption is generally lowered as the gait becomes more symmetric. Currently only powered transtibial powered prostheses have been shown to reduce energy consumption of the user to normal levels during level ground walking. These devices are capable of providing torque/angle characteristics much closer to normal ankle behavior than conventional prostheses (Herr and Grabowski, 2012). But it seems that additional energy asymmetrically injected into the gait cycle could reduce this further (Caputo and Collins, 2014), although at increasingly diminishing returns. This would mean there should be torque/angle characteristics that are more metabolically efficient than the Winter targets for a given walking condition, even if assistance is asymmetrically applied. This is doubly true when considering prosthesis design when the inertial properties of the leg can be custom tailored. In simulation, relaxing the symmetry constraint has shown that it should be possible to reduce amputee cost of transport lower than walking with a biological leg (Handford and Srinivasan, 2016, 2018).

Although symmetric gaits may not be optimal for energy consumption, there is an increased chance of osteoarthritis in the sound leg in transtibial and transfemoral amputees, and there are reasons to believe that increasing joint kinematic symmetry generally leads to reduced detrimental loading, particularly peak force and peak knee external adduction in the contralateral limb (Morgenroth et al., 2011; Grabowski and D'Andrea, 2013). Whether the reduction of these forces actually reduces incidents of osteoarthritis has not been proven, and also it hasn't been proven that restoring torque/angle characteristics of the amputated limb to normal will minimize these forces in a global sense.

Even though natural kinematics and torques do not necessarily minimize metabolic energy consumption or minimize injury, one thing that is certain is that the more normal and symmetric the gait kinematics the more natural and unassuming it looks, which is a large part of the functionality of a daily worn prosthesis. It also provides a familiar starting target for the design of prosthetic limbs which are designed to replace normal limbs. Possibly designs based on non-anthropomorphic principles will allow the discovery of other solutions in the future (LaPrè and Sup, 2013), in much the same way carbon ESR blades revolutionized prostheses for running.

Regardless, the current control of the prosthesis does not attempt to force the Winter kinematics output at the knee and results in an asymmetric gait. This was shown to increase the metabolic rate of the participants (10±9%) when compared to their conventional prostheses. As the subjects become more familiar with the device, the control becomes more refined, and we are able to better apply torques with more accurate timing, we expect an increase in gait symmetry and to eventually reduce metabolic consumption (Malcolm et al., 2013).

#### 5.3.2. Comparison of Kinetics and Kinematics to Normal and C-Leg

**Figure 15:**
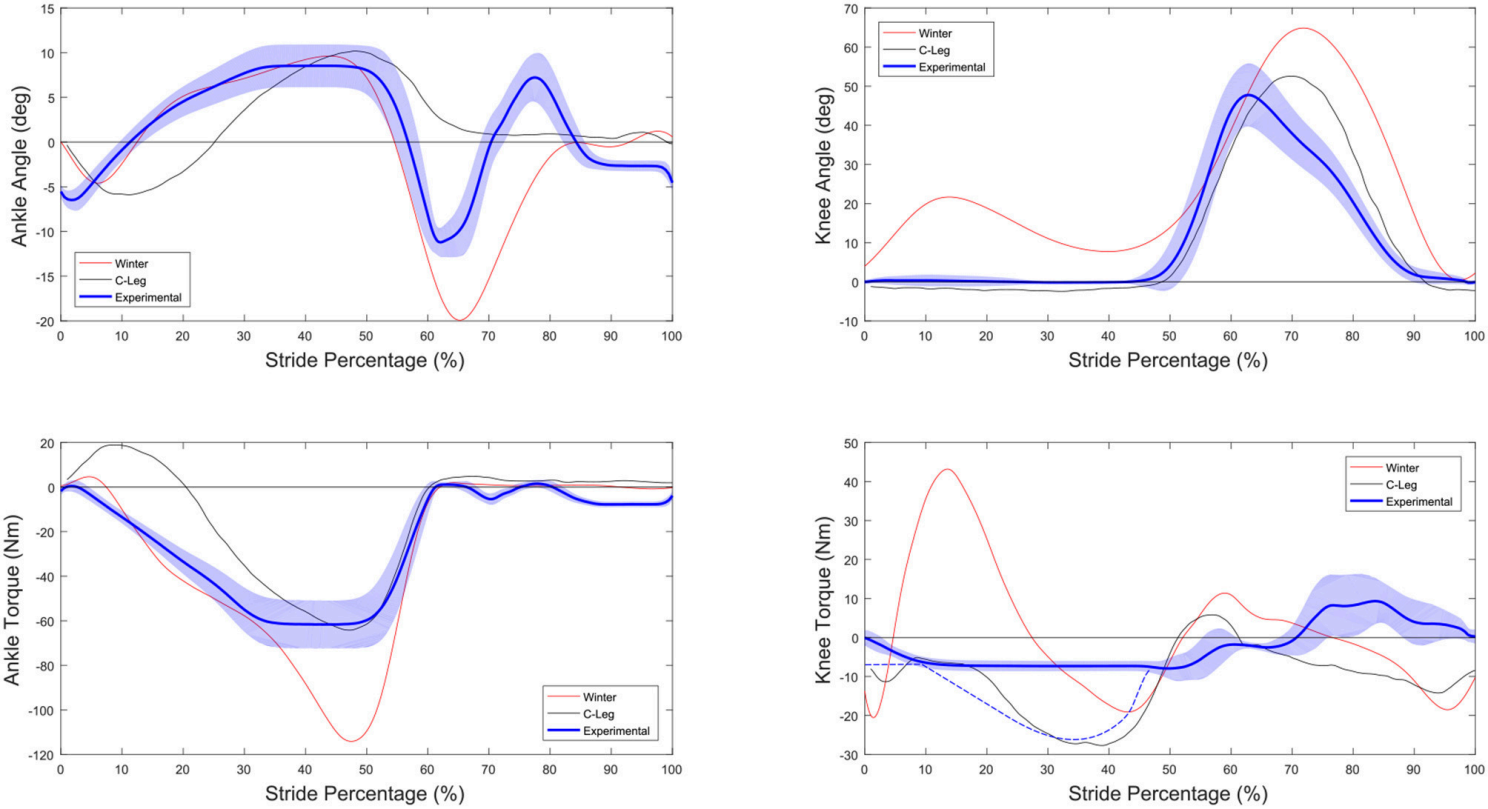
Comparison of the prosthesis behavior vs. the target Winter and C-Leg data (Segal et al., 2006). Kinematics are shown in the upper two graphs while the bottom graphs show the joint torque. Note that because the measurement of joint torque was done using the actuator, the blue dotted line is only an estimate of the knee joint torque based on the behavior of the prosthesis.

Figure 15 shows the knee and ankle torque and kinematics of both the CYBERLEGs prosthesis and the C-Leg as a function of stride percentage, as was done in Figure 13. In these Figures it is a bit clearer to see how the experimental ankle torque essentially followed the biological torque up to the maximum that the subjects requested. It is also clear the ankle had an early pushoff, as well as a large dorsiflexion during the swing phase, both of which were requested by the subjects. The knee joint however has behavior much closer to the microcontroller knee, with the knee remaining on the full extension endstop during the stance phase. Because the knee torque of the prosthesis is measured through the actuator displacement, an estimated blue dotted line has been shown on the knee torque graph which better represents the total knee torque during the stance phase. The major difference between the CYBERLEGs prosthesis and the C-Leg is a powered extension phase at the end of swing phase instead of a braking flexion torque. The subjects felt best knowing the knee would be at full extension at the end of swing phase, presumably because it is difficult to judge how far the knee is bent without visual or sensory feedback such as the leg hitting full extension. They are also familiar and trained to use this method of gait with their current passive prostheses.

#### 5.3.3. Gait Improvements

The current prosthesis control uses motor position setpoints which change the position of the motor side of the SEA based on a heuristic rule-based state machine. This method requires that the dynamic and contact forces of the user are somewhat near to the normal values from which the targets were derived because both the generated kinematics and torques of the joints are completely dependent on these external forces.

There is no feedback of the output trajectory, output impedance, or output torque to compensate for deviation from the target torque/angle in this method. This is actually similar to a rest position microcontroller controlled system, where the rest position of a spring is changed during different phases in the gait cycle, although here the position can be changed while loading and unloading. It was theorized that if the position of the motor side of the spring was placed close enough to the correct position, the loading characteristics of the output could be slightly modified by the walker and they would find the best way to walk with the device, resulting in near normal kinematics and joint torque. In this way, neither the kinematics or the torque are fully constrained. Results show that this tends to work well in the ankle, the users seem to be able to load and unload the ankle in a biologically similar fashion, albeit with reduced energy injection, but with the modified knee control, the knee did not prove to be as well-behaved.

Because the ET was designed to utilize a very specific knee/ankle torque and kinematics relationship, the lack of constraint in the control of the kinematics allowed the device to attain unsafe conditions, and could not be used as designed. It is possible that in a system that is designed to retain angle relationships between the knee and ankle the ET system would work as designed, although because of the actuator effort to keep kinematic accuracy, it isn't guaranteed that this would be useful toward prosthesis energy reduction. Another option would be to determine a gait suitable for an individual without the use of the ET and then adding back ET capability if the gait allows for negative energy of the knee to be transmitted.

When it was decided that the ET portion of the knee could not be used, a new trajectory for the position of the knee carriage was created based on feedback from the people doing the trials, without full regard to the actual torque/angle characteristics of the knee. The subjects also did not use the stance flex WA system, and preferred to utilize the end stop of the knee as much as possible during stance. This behavior may, in part, be to the way conventional sockets are set and how people are trained to use prostheses. Knee hyperextension is often used for knee “stability” during the stance phase using conventional prostheses and it is possible with a modified socket alignment this tendency could be reduced. Control and setup were the main reasons that the behavior of the prosthesis resembled that of a passive prosthesis.

It is clear that a refined, better tuned, control system with clearer goals in system constraints will be required to produce more normal knee torque/angle characteristics. The top level state machine system was not the most adaptable system that could have been chosen for this task, although it was sufficient to obtain preliminary walking gait. Improvements to this will need to include much more training of the user to utilize the WA correctly as well as adding in a better position trajectory of the knee carriage to provide expected knee torque. For topics such as gait symmetry and metabolic consumption, better performance is needed than this control provided. For a fairly complete discussion on different control methods in prostheses and exoskeletons, readers should refer to Tucker et al. (2015) which provides a large array of different methods that may be implemented or examine online optimization methods (Kim et al., 2017; Zhang et al., 2017) to achieve better performance from the control system.

## 6. Conclusions and Future Work

We have created a new, active, combined ankle-knee prosthetic system which achieves as much as possible with a passive approach, using springs that were chosen to match the biological quasi-stiffness of normal gait. These springs are locked and unlocked during the gait cycle and combined with an energy harvesting system to passively provide the majority of the required torque angle characteristics during normal walking, while maintaining versatility by providing active actuation. Under ideal conditions the prosthesis worked on the bench as designed, which also showed a lower motor electrical cost than most current designs. In particular, the capability of the energy transfer system to reduce both the knee and ankle motor work was considerable. In the ideal case the prosthesis performs quite well compared to the current array of powered prostheses.

In reality there are two major issues with the prosthesis. First these savings are all but eliminated by the implementation, where it takes approximately 10 J/stride to capture a similar amount of work in both the WA and ET systems. More efficient, and in the case of the ET more controllable, locking mechanisms should be found to better utilize these systems. Second is that the people wearing the prosthesis do not seem to be able to find walking patterns that utilize the average torque/angle characteristics. Because the prosthesis does not impose either torque or trajectory upon the user, they tend to find gait patterns that are very different from the average biomechanical data. This may be due to training and unfamiliarity with the prosthesis, it may have to do with the nature of the socket interface, inaccuracy of the control timing, or a combination of other reasons. When the user deviates from the average biomechanical trajectories, the energy saving functions of the prosthesis are reduced and the device functions similarly to other powered prostheses under evaluation today.

We conclude with a summary of points learned while developing this prosthesis:

– Bench testing showed the quasi-static stiffness based prosthesis can reproduce average walking knee and ankle joint torques when the output of the prosthesis was constrained with external motors. Under these conditions the ET was found to be capable of transferring energy from the knee to the ankle and a considerable energy consumption reduction of the motors was found.
– The prosthesis was used in a preliminary validation experiment with four amputee subjects and through modification of the main actuator behavior, the prosthesis was able to create a stable gait cycle with all subjects.
– Using the quasi-stiffness estimations from average biomechanical data for the stiffness of the ankle springs creates behaviors that resemble the average biological data in walking trials.
– Using the quasi-stiffness estimations for the stiffness of the knee springs did not provide sufficiently average kinematics and torques during walking trials. Even though it is possible to generate average torque and kinematics in the prosthesis it does not mean the person using it will choose to walk with average torque and kinematics without stabilizing constraints.
– Energy transfer from the knee to the ankle is possible under ideal conditions.
– Because of deviations in the knee and ankle joint kinematics during walking tests, the tests had to be run without the use of the ET system. These mismatches stem from a combination of the prosthesis control, which does not constrain the kinematics, the ET control, which was treated as a locked or unlocked clutch in this implementation, as well as the way the subjects interact with the prosthesis, preferring behaviors that were not like average biomechanical data.
– In order to overcome differences in kinematics, the motor must actuate in a different manner than average biomechanical data would suggest which reduces the efficacy of the quasi-stiffness approach in reducing energy consumption, particularly in the knee.
– The use of low stiffness springs in the knee determined by quasi-stiffness trajectories limit the ability of the actuator to modify the behavior of the knee due to low actuator bandwidth, although solutions can be found that provide stable gait.
– A simple state machine system with a number of experimentally tuned variables to set thresholds for actuation and timing was implemented and work sufficiently to provide basic gait functions. These thresholds were primarily determined by feedback from the patients, and resulted in a powered ankle actuation that was similar to biological ankle function at a reduced amplitude and knee behavior similar to current microcontroller devices.
– It was determined that a much longer training period must be allowed for the users before measurements. Because the prosthesis behaves quite differently to a standard prosthesis, the user must learn to have high trust the device and they must have a detailed understanding of the behavior of the device and how it can be utilized. Training alone may improve kinematics, although it is not the only issue.
– New gait detection and control methods should be able to better utilize the passive aspects of the prosthesis, but how this can best be accomplished is a focus of future work.

## Ethics Statement

This study was carried out in accordance with the recommendations of Fondazione Don Carlo Gnocchi. Ethical approval for the protocol was obtained through FDG. All subjects gave written informed consent in accordance with the Declaration of Helsinki.

## Author Contributions

LF designed and constructed the hardware, conducted experiments, analyzed data, and wrote the manuscript. JG designed and constructed the hardware, conducted experiments, analyzed data, and proofread the manuscript. RJ-F supervised experiments at VUB. SH helped with data analysis and derived governing torque equations. BV supervised experiments at VUB and proofread the manuscript. MM provided the WSA system and assisted with top level control. RM presided over the experimental sessions at FDG, gained ethical approvals, and lead patient recruitment. NV supervised the experimental sessions. DL supervised the hardware design and proofread the manuscript.

### Conflict of Interest Statement

The authors declare that the research was conducted in the absence of any commercial or financial relationships that could be construed as a potential conflict of interest.

## References

[B1] Ambrozic L. Gorsic M. Geeroms J. Flynn L. Molino Lova R. Kamnik R. (2014). Cyberlegs: a user-oriented robotic transfemoral prosthesis with whole-body awareness control. Rob. Autom. Mag. IEEE 21, 82–93. 10.1109/MRA.2014.2360278

[B2] Au S. Herr H. (2008). Powered ankle-foot prosthesis: the importance of series and parallel motor elasticity. IEEE Rob. Autom. Mag. 15, 52–59. 10.1109/MRA.2008.927697

[B3] Caputo J. M. Collins S. H. (2014). Prosthetic ankle push-off work reduces metabolic rate but not collision work in non-amputee walking. Sci. Rep. 4:7213 10.1038/srep07213 25467389 PMC4252906

[B4] Cuccurullo S. (ed.). (2004). Physical Medicine and Rehabilitation Board Review. New York, NY: Demos.

[B5] Flynn L. Geeroms J. Jimenez-Fabian R. Vanderborght B. Vitiello N. Lefeber D. (2015). Ankle-knee prosthesis with active ankle and energy transfer: Development of the CYBERLEGs Alpha-Prosthesis. Rob. Auton. Syst. 73, 4–15. 10.1016/j.robot.2014.12.013

[B6] Flynn L. L. Geeroms J. van der Hoeven T. Vanderborght B. Lefeber D. (2018). Vub-cyberlegs cybathlon 2016 beta-prosthesis: case study in control of an active two degree of freedom transfemoral prosthesis. J. Neuroeng. Rehabil. 15:3 10.1186/s12984-017-0342-y 29298695 PMC5751827

[B7] Garate V. R. Parri A. Yan T. Munih M. Lova R. M. Vitiello N. (2016). Walking assistance using artificial primitives: a novel bioinspired framework using motor primitives for locomotion assistance through a wearable cooperative exoskeleton. IEEE Rob. Autom. Mag. 23, 83–95. 10.1109/MRA.2015.2510778

[B8] Geeroms J. Flynn L. Jimenez-Fabian R. Vanderborght B. Lefeber D. (2017). Design and energetic evaluation of a prosthetic knee joint actuator with a lockable parallel spring. Bioinspiration Biomimetics 12:026002 10.1088/1748-3190/aa575c 28059775

[B9] Geeroms J. Flynn L. Jimenez-Fabian R. Vanderborght B. Lefeber D. (2018). Energetic analysis and optimization of a maccepa actuator in an ankle prosthesis. Auton. Rob. 42, 147–158. 10.1007/s10514-017-9641-1

[B10] Genin J. J. Bastien G. J. Franck B. Detrembleur C. Willems P. A. (2008). Effect of speed on the energy cost of walking in unilateral traumatic lower limb amputees. Eur. J. Appl. Physiol. 103, 655–663. 10.1007/s00421-008-0764-0 18478251

[B11] Gilbert B. Lambry D. (2013). Joint Actuation Mechanism for a Prosthetic and/or Orthotic Device Having a Compliant Transmission. Patent No. US8435309. Available online at: https://patents.google.com/patent/US8435309

[B12] Giovacchini F. Vannetti F. Fantozzi M. Cempini M. Cortese M. Parri A. (2015). A light-weight active orthosis for hip movement assistance. Rob. Auton. Syst. 73, 123–134. 10.1016/j.robot.2014.08.015

[B13] Goršic M. Kamnik R. Ambrožic L. Vitiello N. Lefeber D. Pasquini G.. (2014). Online phase detection using wearable sensors for walking with a robotic prosthesis. Sensors 14, 2776–2794. 10.3390/s140202776 24521944 PMC3958303

[B14] Grabowski A. M. D'Andrea S. (2013). Effects of a powered ankle-foot prosthesis on kinetic loading of the unaffected leg during level-ground walking. J. Neuroeng. Rehabil. 10, 1–12. 10.1186/1743-0003-10-49 23758860 PMC3685554

[B15] Hafner B. J. Sanders J. E. Czerniecki J. M. Fergason J. (2002). Transtibial energy-storage-and-return prosthetic devices: a review of energy concepts and a proposed nomenclature. Bull. Prosthet. Res. 39, 1–11. Available online at: https://www.rehab.research.va.gov/jour/02/39/1/pdf/hafner.pdf 11926321

[B16] Handford M. L. Srinivasan M. (2016). Robotic lower limb prosthesis design through simultaneous computer optimizations of human and prosthesis costs. Nat. Sci. Rep. 6:19983. 10.1038/srep19983 26857747 PMC4746571

[B17] Handford M. L. Srinivasan M. (2018). Energy-optimal human walking with feedback-controlled robotic prostheses: A computational study. IEEE Trans. Neural Syst. Rehabil. Eng. 26, 1773–1782. 10.1109/TNSRE.2018.2858204 30040647

[B18] Heins S. Flynn L. Geeroms J. Lefeber D. Ronsse R. (2018). Torque control of an active elastic transfemoral prosthesis via quasi-static modelling. Rob. Auton. Syst. 107, 100–115. 10.1016/j.robot.2018.05.015

[B19] Herr H. M. Grabowski A. M. (2012). Bionic ankle-foot prosthesis normalizes walking gait for persons with leg amputation. Proc. R. Soc. Lon. B 279, 457–464. 10.1098/rspb.2011.1194 21752817 PMC3234569

[B20] Hitt J. K. Bellman R. Holgate M. Sugar T. G. Hollander K. W. (2008). The sparky (spring ankle with regenerative kinetics) project: design and analysis of a robotic transtibial prosthesis with regenerative kinetics, in 2007 Proceedings of the ASME International Design Engineering Technical Conferences and Computers and Information in Engineering Conference, DETC2007, vol. 5 PART C (Las Vegas, NV), 1587–1596.

[B21] Jimenez-Fabian R. Flynn L. Geeroms J. Vitiello N. Vanderborght B. Lefeber D. (2015). Sliding-Bar MACCEPA for a powered ankle prosthesis. J. Mech. Rob. 7, 1–2. 10.1115/1.4029439

[B22] Jimenez-Fabian R. Geeroms J. Flynn L. Vanderborght B. Lefeber D. (2017). Reduction of the torque requirements of an active ankle prosthesis using a parallel spring. Rob. Auton. Syst. 92, 187–196. 10.1016/j.robot.2017.03.011

[B23] Kim M. Ding Y. Malcolm P. Speeckaert J. Siviy C. J. Walsh C. J.. (2017). Human-in-the-loop bayesian optimization of wearable device parameters. PLoS ONE 12:e0184054. 10.1371/journal.pone.0184054 28926613 PMC5604949

[B24] LaPrè A. K. Sup F. (2013). Redefining prosthetic ankle mechanics: Non-anthropomorphic ankle design, in 2013 IEEE 13th International Conference on Rehabilitation Robotics (ICORR) (Seattle, WA), 1–5. 10.1109/ICORR.2013.6650439 24187257

[B25] Lawson B. E. Mitchell J. Truex D. Shultz A. Ledoux E. Goldfarb M. (2014). A robotic leg prosthesis: design, control, and implementation. IEEE Rob. Autom. Mag. 21, 70–81. 10.1109/MRA.2014.2360303

[B26] Lenzi T. Cempini M. Hargrove L. Kuiken T. (2018). Design, development, and testing of a lightweight hybrid robotic knee prosthesis. Int. J. Rob. Res. 37, 953–976. 10.1177/0278364918785993

[B27] Lenzi T. Sensinger J. Lipsey J. Hargrove L. Kuiken T. (2015). Design and preliminary testing of the RIC hybrid knee prosthesis. in Proceedings of the Annual International Conference of the IEEE Engineering in Medicine and Biology Society, EMBS (Milan), 1683–1686. 10.1109/EMBC.2015.7318700 26736600

[B28] Malcolm P. Derave W. Galle S. De Clercq D. (2013). A simple exoskeleton that assists plantarflexion can reduce the metabolic cost of human walking. PLoS ONE 8:e56137. 10.1371/journal.pone.0056137 23418524 PMC3571952

[B29] Matthys A. Cherelle P. Van Damme M. Vanderborght B. Lefeber D. (2012). Concept and design of the hekta (harvest energy from the knee and transfer it to the ankle) transfemoral prosthesis, in 2012 4th IEEE RAS EMBS International Conference on Biomedical Robotics and Biomechatronics (BioRob) (Rome), 550–555.

[B30] Mauch H. (1968). Stance control for above-knee artificial legs-design considerations in the s-n-s knee. Bull. Prosthet. Res. 20, 61–72.

[B31] Morgenroth D. C. Segal A. D. Zelik K. E. Czerniecki J. M. Klute G. K. Adamczyk P. G.. (2011). The effect of prosthetic foot push-off on mechanical loading associated with knee osteoarthritis in lower extremity amputees. Gait Posture 34, 502–507. 10.1016/j.gaitpost.2011.07.001 21803584 PMC3189331

[B32] Parri A. Martini E. Geeroms J. Flynn L. Pasquini G. Crea S. (2017). Whole body awareness for controlling a robotic transfemoral prosthesis. Front. Neurorob. 11:25 10.3389/fnbot.2017.00025 PMC5448151 28611621

[B33] Pfeifer S. Pagel A. Riener R. Vallery H. (2014). Actuator with angle-dependent elasticity for biomimetic transfemoral prostheses. IEEE/ASME Trans. Mechatronics 20, 1384–1394. 10.1109/TMECH.2014.2337514

[B34] Plooij M. Mathijssen G. Cherelle P. Lefeber D. Vanderborght B. (2015). Lock your robot: a review of locking devices in robotics. IEEE Rob. Autom. Mag. 22, 106–117. 10.1109/MRA.2014.2381368

[B35] Pratt J. Krupp B. Morse C. (2002). Series elastic actuators for high fidelity force control. Ind. Robot 29, 234–241. 10.1108/01439910210425522

[B36] Ronsse R. De Rossi S. Vitiello N. Lenzi T. Carrozza M. Ijspeert A. (2013). Real-time estimate of velocity and acceleration of quasi-periodic signals using adaptive oscillators. Rob. IEEE Trans. 29, 783–791. 10.1109/TRO.2013.2240173

[B37] Rouse E. J. Mooney L. M. Herr H. M. (2014). Clutchable series-elastic actuator: implications for prosthetic knee design. Int. J. Rob. Res. 33, 1611–1625. 10.1177/0278364914545673

[B38] Ruiz Garate V. Parri A. Yan T. Munih M. Molino Lova R. Vitiello N. (2017). Experimental validation of motor primitive-based control for leg exoskeletons during continuous multi-locomotion tasks. Front. Neurorob. 11:15 10.3389/fnbot.2017.00015 PMC5355439 28367121

[B39] Segal A. D. Orendurff M. S. Klute G. K. McDowell M. L. Pecoraro J. A. Shofer J. (2006). Kinematic and kinetic comparisons of transfemoral amputee gait using c-leg® and mauch sns® prosthetic knees. J. Rehabil. Res. Dev. 43, 857 10.1682/JRRD.2005.09.0147 17436172

[B40] Shamaei K. Sawicki G. S. Dollar A. M. (2013). Estimation of quasi-stiffness and propulsive work of the human ankle in the stance phase of walking. PLoS ONE 8:e59935. 10.1371/journal.pone.0059935 23555839 PMC3605342

[B41] Staros A. (1957). The sach (solid-ankle cushion-heel) foot. Orthop. Prosth. Appl. J. 11, 23–31.

[B42] Sup F. Varol H. A. Mitchell J. Withrow T. J. Goldfarb M. (2009). Preliminary evaluations of a self-contained anthropomorphic transfemoral prosthesis. IEEE/ASME Trans. Mechatr. 14, 667–676. 10.1109/TMECH.2009.2032688 20054424 PMC2801882

[B43] Tucker M. R. Olivier J. Pagel A. Bleuler H. Bouri M. Lambercy O.. (2015). Control strategies for active lower extremity prosthetics and orthotics: a review. J. Neuroeng. Rehabil. 12, 1–29. 10.1186/1743-0003-12-1 25557982 PMC4326520

[B44] Unal R. Klijnstra F. Burkink B. Behrens S. M. Hekman E. E. G. Stramigioli S. (2013). Modeling of walkmech: a fully-passive energy-efficient transfemoral prosthesis prototype, in Rehabilitation Robotics (ICORR), 2013 IEEE International Conference on (Seattle, WA), 1–6. 10.1109/ICORR.2013.6650406 24187225

[B45] Van Ham R. Vanderborght B. van Damme M. Verrelst B. Lefeber D. (2007). MACCEPA, the mechanically adjustable compliance and controllable equilibrium position actuator: Design and implementation in a biped robot. Rob. Auton. Syst. 55, 761–768. 10.1016/j.robot.2007.03.001

[B46] Winter D. A. (2009). Biomechanics and Motor Control of Human Movement, 4th Edn Hoboken, NJ: John Wiley and Sons.

[B47] Zhang J. Fiers P. Witte K. A. Jackson R. W. Poggensee K. L. Atkeson C. G.. (2017). Human-in-the-loop optimization of exoskeleton assistance during walking. Science 356, 1280–1284. 10.1126/science.aal5054 28642437

