# Supplementary material for: The Challenges and Achievements of Experimental Implementation of an Active Transfemoral Prosthesis Based on Biological Quasi-Stiffness: The CYBERLEGs Beta-Prosthesis
Source: Front Neurorobot. 2018 Dec 4;12:80. doi: 10.3389/fnbot.2018.00080 (PMC6289037; doi:10.3389/fnbot.2018.00080)
Supplement: Supplementary file 2 [file Data_Sheet_1.pdf]

The Challenges and Achievements of Experimental Implementation of an Active Transfemoral Prosthesis Based on Biological Quasi-Stiffness: The CYBERLEGs Beta-Prosthesis

Louis Flynn [1,*], Joost Geeroms [1], Rene Jimenez-Fabian [1], Sophie Heins [2], Bram Vanderborght [1], Marko Munih [3], Raffaele Molino Lova [4], Nicola Vitiello [4,5], and Dirk Lefeber [1]

[1] *Robotics and Multibody Mechanics Department, Vrije Universiteit Brussel, and Flanders Make, Brussels, Belgium*
[2] *Center for Research in Mechatronics; Institute of Mechanics, Materials, and Civil Engineering; Institute of Neuroscience; and Louvain Bionics; Université catholique de Louvain, Louvain-la-Neuve, Belgium*
[3] *Robolab, Faculty of Electrical Engineering, University of Ljubljana, Ljubljana, Slovenia*
[4] *Fondazione Don Carlo Gnocchi, Firenze, Italy*
[5] *The BioRobotics Institute, Scuola Superiore Sant'Anna, Pisa, Italy*

Correspondence*:
Louis Flynn
lflynn@vub.ac.be

1 APPENDIX

The kinematic equations governing the quasi-static torque of the prosthesis joints are summarized in this section. For a more complete version of these derivations, please refer to **?**.

1.1 Ankle Kinematics

The ankle torque at any point in time, (T_A), is a combination of the MACCEPA actuator, parallel spring torque, and the torque caused by the ET system T^a_{ET}.

$$T_A(\alpha, P) = C(\alpha) f(\alpha, P) + T_{Parallel} + T^a_{ET} \tag{1}$$

Which is the product of the distance along the foot shaft, C, defined as

$$C(\alpha) = B\cos\alpha + A\left[1 - \left(\frac{B}{A}\sin\alpha\right)^2\right]^{1/2} \tag{2}$$

and the perpendicular force to the foot shaft, $f(\alpha, P)$, defined as

$$f(\alpha,P) = \frac{kB(P+A+B-C(\alpha))\sin\alpha}{A\left[1-\left(\frac{B}{A}\sin\alpha\right)^2\right]^{1/2}} \tag{3}$$

where $f(\alpha,P)$ is the force acting on b perpendicular to $\overline{ab}$. Note that $B/A<1$ should be satisfied to avoid a singularity at $\alpha = 90deg$.

to which the parallel spring torque, $T_{Parallel}$, is added which can be approximated by the displacement of the ankle in plantarflexion with respect to the rest position of the parallel spring, which in this case was approximately -3.5 degrees and stiffness of 6 Nm/deg.

$$T_{Parallel}\begin{cases}\approx 6*(\theta_A-\theta_{parallel}) \text{ if } \theta_A < -3.5\\ = 0; \text{ if } \theta_{ankle} > -3.5\end{cases}$$

1.2 Weight Acceptance Kinematics

The WA system, shown in Figure **??**, provides a knee joint torque when the knee is flexed past the rest angle of the WA spring. This angle is determined by the nut position, Z_{WA}, which is driven by the WA motor. The length of the WA is determined by

$$X_{WA} = \sqrt{C_k^2 + Y_{WA}^2 - 2*C_k*Y_{WA}*\cos(\pi-\lambda-\theta_k)} \tag{4}$$

and the knee torque from the WA is then

$$T_{WA} = C_k * K_{WA} * (X_{WA}^0 - X_{WA})\sin(\epsilon) \tag{5}$$

where X_{WA}^0 is the rest length of the WA. Through substitution of ϵ, the resulting knee torque is

$$T_{WA} = \frac{C_k * Y_{WA} * K_{WA} * (X_{WA}^0 - X_{WA})\sin(\pi-\lambda-\theta_k)}{X_{WA}} \tag{6}$$

1.3 Knee Actuator Kinematics

The knee actuator schematic is shown on the right side of Figure **??**. The torque around the knee joint, a, due to the actuator is determined by the equation

$$T_{KA} = F_{res}A_k\cos(\gamma_c)*\big(\cos(\theta_k-\alpha_k)-\sin(\theta_k-\alpha_k)\tan(\xi-\gamma_c)\big) \tag{7}$$

where F_{res} is the spring force on the pushrod B_k in the direction of the carriage rail $\bar{bc}$. A_k, γ_c, and α_k are geometric properties of the prosthesis, and ξ is defined as

$$\xi = \sin^{-1}\Big(\frac{1}{B_K}\big(X_c \sin(\frac{\pi}{2} - \gamma_c) - A_k \sin(\frac{\pi}{2} - \gamma_c + \alpha_k - \theta_k)\big)\Big) \tag{8}$$

The position of the carriage relative to the end of the pushrod determines which spring is active. To calculate this let

$$u = Z_0 - Z_{ka} - \frac{L_2}{\cos(\gamma_c)} \tag{9}$$

where Z_0 is the position of the carriage with zero torque when the knee is fully extended ($\theta_k = 0$) and Z_{ka} is the position of the carriage commanded by the motor.

L_1 and L_2 are the horizontal and vertical displacements from the knee joint of the end of the pushrod. These can be determined to be

$$L_1 = A_k \cos(\alpha_k - \theta_k) + B_k \sin(\xi - \gamma_c) \tag{10}$$

$$L_2 = A_k \sin(\alpha_k - \theta_k) + B_k \cos(\xi - \gamma_c) \tag{11}$$

Then

$$F_{res} = \begin{cases} F_{BL} & \text{if } u < D - L_{EX}^0 \\ -F_{EX} & \text{if } u > L_{BL}^0 \\ F_{BL} - F_{EX} & \text{otherwise} \end{cases}$$

where D is the carriage length, L_{EX}^0 and L_{BL}^0 are the initial rest lengths of the extension and baseline spring, respectively, and

$$F_{BL} = K_{BL}(L_{BL}^0 - u) \tag{12}$$

$$F_{EX} = K_{EX}(L_{EX}^0 - D - u). \tag{13}$$

1.4 Energy Transfer Kinematics

The torque on the knee due to the ET is

$$T_{ET}^k = F_{ET}(L_1 \cos(\epsilon) + L_2 \sin(\epsilon)) \tag{14}$$

where

$$\epsilon = \tan^{-1}\left(\frac{X_c - p_{1x} - L_2 \tan(\gamma_c)}{L_{sh} - p_{1y} - L_2}\right) \tag{15}$$

and L_1 and L_2 are defined as Equations 10 and 11 and p_{1x} and p_{1y} are defined as the position of pulley d with respect to the ankle joint.

The torque on the ankle joint due to the ET is

$$T^a_{ET} = F_{ET} * E * \cos(\theta_a - \zeta_{ET} + \frac{\pi}{2} + \delta) \tag{16}$$

where

$$\delta = \tan^{-1}\left(\frac{E * \cos(\theta_a - \zeta_{ET} + \frac{\pi}{2}) - p_{2x}}{p_{2y} + E * \sin(\theta_a - \zeta_{ET} + \frac{\pi}{2})}\right) \tag{17}$$

The force in the ET cable is

$$F_{ET} = K_{ET}(L_{ET} - L^0_{ET}) \tag{18}$$

where K_{ET} is the spring constant of the ET cable, L^0_{ET} is the initial locking of the ET system, and L_{ET} is defined as

$$L_{ET} = d_1 + d_2 + d_3 - L_c \tag{19}$$

where L_c is the length of the ET cable and

$$d_1 = 2\sqrt{(L_{sh} - p_{1y} - L_2)^2 + (X_c - p_{1x} - L_2 \tan(\gamma_c))^2} \tag{20}$$

$$d_2 = \sqrt{(p_{2y} + E * \sin(\theta_{ET}))^2 + (p_{2x} - E * \cos(\theta_{ET}))^2} \tag{21}$$

$$d_3 = \sqrt{(p_{1x} + p_{2x})^2 + (p_{2y} - p_{1y})^2} \tag{22}$$

and if $L_{ET} > L^0_{ET}, F_{ET} = 0$ due to the fact that the ET cable would be slack in this condition.
